# A Census of Marine Biodiversity Knowledge, Resources, and Future Challenges

**DOI:** 10.1371/journal.pone.0012110

**Published:** 2010-08-02

**Authors:** Mark John Costello, Marta Coll, Roberto Danovaro, Pat Halpin, Henn Ojaveer, Patricia Miloslavich

**Affiliations:** 1 Leigh Marine Laboratory, University of Auckland, Warkworth, New Zealand; 2 Dalhousie University, Halifax, Canada; 3 Institute of Marine Science (ICM-CSIC), Barcelona, Spain; 4 Department of Marine Sciences, Polytechnic University of Marche, Ancona, Italy; 5 Nicholas School of the Environment, Duke University, Durham, North Carolina, United States of America; 6 Estonian Marine Institute, University of Tartu, Pärnu, Estonia; 7 Departamento de Estudios Ambientales and Centro de Biodiversidad Marina, Universidad Simón Bolívar, Caracas, Venezuela; University of Hull, United Kingdom

The Census of Marine Life (2000–2010) was the largest global research programme on marine biodiversity. This paper integrated the findings of reviews of major world regions by the Census and provides a global perspective on what is known and what are the major scientific gaps. Study metrics were regional species richness, numbers of endemic and alien species, numbers of species identification guides and taxonomic experts, and a state-of-knowledge index. The threats to biodiversity were classified across the regions. A poor to moderate correlation between species richness and seabed area, and sea volume, and no correlations with topographic variation, were attributed to sparse, uneven and unrepresentative sampling in much of the global marine environment. Many habitats have been poorly sampled, particularly in deeper seas, and several species-rich taxonomic groups, especially of smaller organisms, remain poorly studied. Crustacea, Mollusca, and Pisces comprised approximately half of all known species across the regions. The proportion that these and other taxa comprised of all taxa varied sufficiently to question whether the relative number of species within phyla and classes are constant throughout the world. Overfishing and pollution were identified as the main threats to biodiversity across all regions, followed by alien species, altered temperature, acidification, and hypoxia, although their relative importance varied among regions. The findings were replicated worldwide, in both developed and developing countries: i.e. major gaps exist in sampling effort and taxonomic expertise that impair society's ability to discover new species and identify and understand species of economic and ecological importance. There was a positive relationship between the availability of species identification guides and knowledge of biodiversity, including the number of species and alien species. Available taxonomic guides and experts correlated negatively with endemic species, suggesting that the more we study the ocean the fewer endemic species are evident. There is a need to accelerate the discovery of marine biodiversity, since much of it may be lost without even being known. We discuss how international collaboration between developed and developing countries is essential for improving productivity in the discovery and management of marine biodiversity, and how various sectors may contribute to this.

## Introduction

The resources available for research are always limited. When setting priorities for research funding, governments, industry, and funding agencies must balance the demands of human health, food supply, and standards of living, against the less-tangible benefits of discovering more about the planet's biodiversity. Scientists have discovered almost 2 million species indicating that we have made great gains in our knowledge of biodiversity. However, this knowledge may distract attention from the estimated four-fifths of species on Earth that remain unknown to science, many of them inhabiting our oceans [1], [2]. The world's media still find it newsworthy when new species are discovered [1]. However, the extent of this taxonomic challenge no longer appears to be a priority in many funding agencies, perhaps because society and many scientists believe we have discovered most species, or that doing so is out of fashion except when new technologies are employed. Another symptom of this trend may be that the increased attention to novel methods available in molecular sciences is resulting in a loss of expertise and know-how in the traditional descriptive taxonomy of species [3]. The use of molecular techniques complements traditional methods of describing species but has not significantly increased the rate of discovery of new species (at least of fish), although it may help classify them [4]. At least in Europe, there was a mismatch between the number of species in a taxon and the number of people with expertise in it [5]. Unfortunately, because most species remain to be discovered in the most species-rich taxa [2], [5], [6], [7], there are then few experts to appreciate that this work needs to be done. Evidently, a global review of gaps in marine biodiversity knowledge and resources is overdue.

### History of discovering marine biodiversity

Although the economic exploitation of marine resources dates back to prehistoric times, and historical documentation has existed since the third century B.C. with Aristotle's contributions in the Mediterranean Sea (e.g. [8]), the establishment of systematic collections of marine organisms began only during the seventeenth and eighteenth centuries. Global marine biodiversity investigations at these times depended not only on the availability of expertise, but also on foreign policies of the colonial powers of the time. For those reasons, the specimens collected from several regions (e.g., Caribbean, Japan, South America, Africa) were mostly brought to Europe, where they were described, deposited in museum collections, and used for the production of marine biological monographs. These early publications contained descriptions and checklists of many marine species, such as molluscs, crustaceans, fishes, turtles, birds, and mammals (e.g. [9], [10], [11]).

The history of research on marine biodiversity can generally be divided into three periods: early exploratory studies, local coastal “descriptive” studies, and large-scale multidisciplinary investigations and syntheses. These periods vary in timing by different seas and countries. The first exploratory studies in several regions (e.g., South America, Caribbean, South Africa, Pacific Ocean) took place from the mid-1700s until the late-1800s, in association with mainly European, North American, and Russian exploration expeditions, such as the Kamchatka Expedition in the 1740s, James Cook's voyages in the 1770s, the cruise of HMS *Beagle* in the 1830s, the voyage of HMS *Challenger* in the 1870s, and the first deep-sea investigations in the Mediterranean Sea [8], [9], [12], [13]. Pioneer investigations on deep-sea organisms were conducted in the Aegean Sea, where Forbes [14] noticed that sediments became progressively more impoverished in terms of biodiversity with increasing sampling depth. The azoic hypothesis proposed by Forbes suggested that life would be extinguished beyond 500 m depth, although a work published 68 years earlier provided indisputable evidence of the presence of life in the Gulf of Genoa at depths down to 1,000 m [15].

The taxonomists who described marine species at these times seldom collected specimens themselves in the field and, therefore, had only second-hand information about the distribution and ecology of the samples they received [4], [8]. Some of the early descriptions of tropical species thus do not even have the locality where the holotype or voucher material was collected (some examples in Chenu 1842–1853). The second period of regional studies was initiated by enhanced availability of research resources (experts, institutes, and vessels) in developing countries around the mid-1900s. The earliest institutions and research stations, many of which continue to operate, were founded in some areas as early as the late 1800s and early 1900s (e.g. [11], [16], [17]). Wide-scale establishment of laboratories in several continents (Europe, New Zealand, North and South America) have only been operational since the 1950s–1960s. The third stage, large-scale multidisciplinary investigations, has evolved since the 1990s, and is related to development and application of modern technologies and implementation of large, multinational research projects. Perhaps the largest of such investigations was the Census of Marine Life (Census).

### The Census of Marine Life

The Census has been the largest-ever, worldwide collaboration of marine biologists, involving more than 2,700 scientists from more than 80 countries and many other collaborators [18]. It spanned the decade of 2000–2010, involved some 538 field expeditions, cost US$650 million, and discovered at least 1,200 species new to science; some specimen collections are still being analysed, so more species new to science will be described. The Census has produced more than 2,600 publications already and generated 24 worldwide media releases that were taken up by over a thousand media outlets (including TV and radio, as well as printed and online media) in at least 50 languages in 57 countries [19], and popular books [20], [21]. The Census was organised into field exploration projects, online database publication, and projects that analysed past and predicted future scenarios for marine biodiversity. It also established National and Regional Implementation Committees (NRIC) to aid coordination of activities. These regional committees came together through national and regional workshops, resulting in the publication of several local or regional journals or books about the state of knowledge of marine biodiversity in their regions [22]. During this decade of Census activities, the committees benefited from Census field exploration and data gathering projects, as well as other national and regional initiatives aimed to enhance the knowledge on marine biodiversity. The committee's findings have been published in detailed reviews of current knowledge and resources in this journal. This paper provides a synthesis of their findings and compares what we know now about marine biodiversity in different geographic regions of the world. It explores how this knowledge is related to what resources and expertise occur in these regions, and provides recommendations of how the major research challenges may be addressed in the next decade.

## Methods

**Figure 1:**
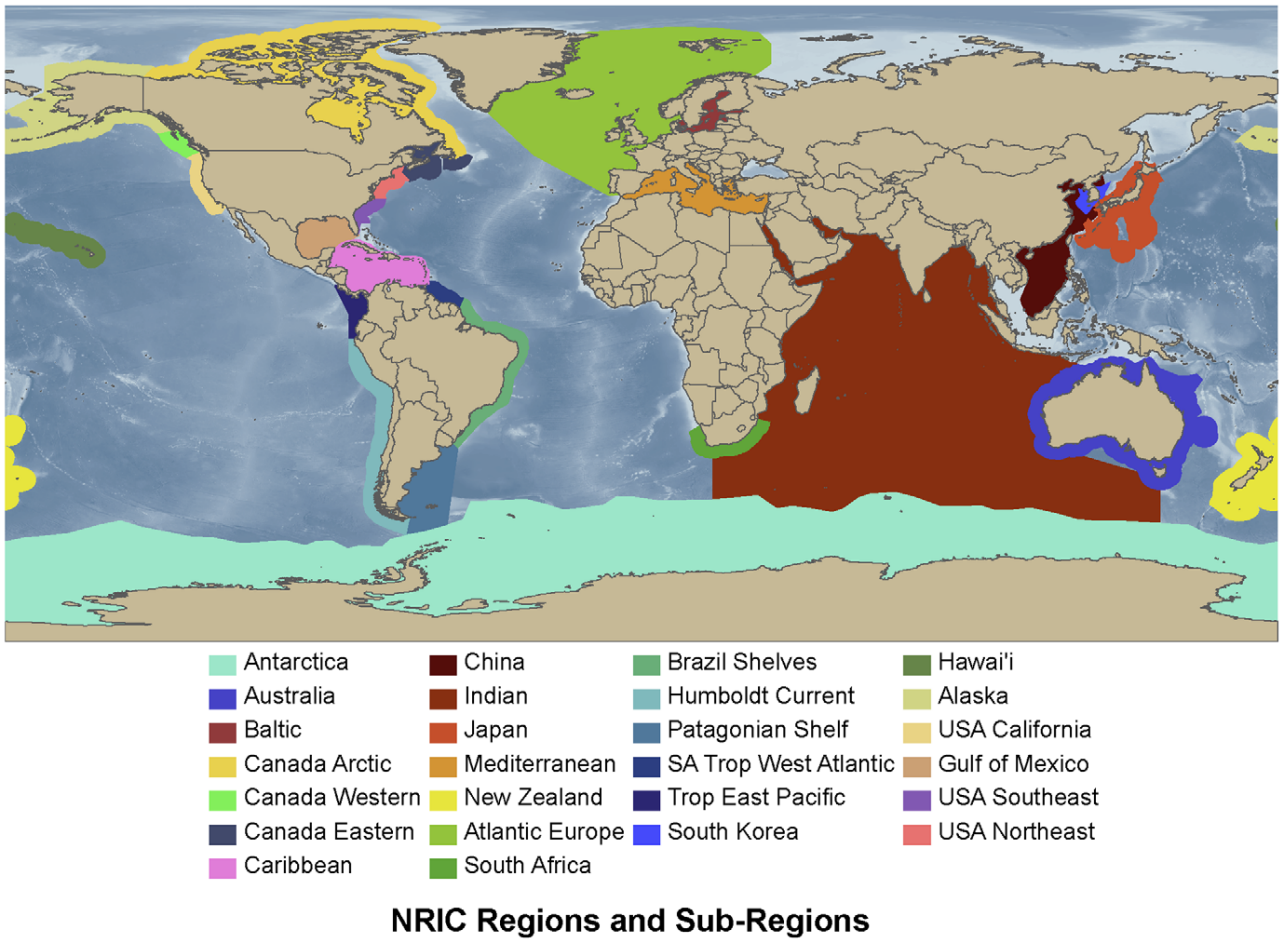
The location of the geographic regions reviewed by the Census of Marine Life (**Table 1**).

The Census NRIC together comprised over 360 scientists from many institutions. Their collective knowledge, including published and unpublished data from within their region, were brought together to review what was known about marine biodiversity in their region (Table 1, Figure 1). These regions were Antarctica [16], Atlantic Europe [15], Australia [23], Baltic Sea [24], Canada [25], Caribbean Sea [9], China [26], Indian Ocean [27], Japan [9], Mediterranean Sea [8], [28], New Zealand [29], South Africa [12], South America [10], South Korea [30], and the USA [11]. These papers provided the data used here. Because every NRIC was not able to provide all the categories of data analysed here, not every region is represented in every table and graph.

**Table 1:** The NRIC regions seabed area and volume, total eukaryote species richness, and richness per area (multiplied by 1,000 for presentation purposes).

NRIC region	No. species	Seabed area km^2^	Sea volume km^3^	spp/area
Alaska^1^	5,925	3,654,304	8,666,714	1.6
Antarctica^3^	8,200	21,186,153	70,628,284	0.4
Atlantic Europe^4^	12,270	3,572,655	4,553,917	3.4
Australia^1^	32,889	6,819,501	15,272,583	4.8
Baltic^5^	5,865	411,218	26,353	14.3
Brazil shelves^2^	9,101	2,520,420	6,797,196	3.6
Canada Arctic^2^	3,038	3,233,113	2,769,789	0.9
Canada Eastern^2^	3,160	823,799	705,744	3.8
Canada Western^2^	2,636	317,363	271,883	8.3
Caribbean^3^	12,046	2,828,125	7,219,167	4.3
China^1^	22,365	831,966	66,825	26.9
Gulf of Mexico^3^	15,374	1,518,067	2,344,179	10.1
Hawaii^1^	8,244	2,459,609	11,212,445	3.4
Humboldt Current^2^	10,186	3,127,380	8,434,076	3.3
Japan^1^	32,777	3,970,743	14,721,516	8.3
Mediterranean^6^	16,848	2,451,059	3,833,673	6.9
New Zealand^1^	12,780	4,073,895	10,004,545	3.1
Patagonian Shelf^2^	3,776	2,693,614	7,264,273	1.4
SA Trop West Atlantic^2^	2,743	604,068	1,629,080	4.5
South Africa^1^	12,915	846,463	1,758,244	15.3
South Korea^1^	9,900	306,674	166,752	32.3
Trop East Pacific^2^	6,696	905,540	2,442,107	7.4
USA California^2^	10,160	1,053,172	1,933,718	9.6
USA Northeast^2^	5,045	692,073	1,270,708	7.3
USA Southeast^2^	4,229	624,984	1,147,525	6.8

Data sources cited in Methods. SA  =  South America (excluding Caribbean coasts); Trop  =  tropical. Spatial statistics based on (1) Exclusive Economic Zone, (2) portion of all EEZ for South America, USA, or Canada, (3) sea area, (4) combination of Norwegian, North, Irish, Greenland, and Celtic seas; Bay of Biscay; English, St. Georges, and Bristol channels; Inner Seas off West Scotland, (5) combination of Baltic Sea, Kattegat, Gulf of Bothnia, Gulf of Finland, Gulf of Riga, and (6) combination of Mediterranean Sea, Tyrrhenian Sea, Aegean Sea, Ionian Sea, Adriatic Sea, Ligurian Sea, Strait of Gibraltar, Alboran Sea [31].

The number of eukaryote species per taxon was used as the basic metric of biodiversity knowledge. Other aspects of biodiversity, such as within-species and ecosystem levels of diversity, build on such species knowledge. Because a different metric of prokaryote diversity is required than the species concepts as applied to eukaryotes, we did not quantify prokaryote diversity, although some regional syntheses provided estimates and comments on the state of knowledge about prokaryote diversity (e.g. [8], [10], [11]). The NRIC derived estimates of their species richness from the literature, databases, and opinions of their regional taxonomic experts.

Here we investigated the collective knowledge assembled by the NRIC and correlated species richness with seabed area, volume, and an index of topographic variation from data [31]. The topographic index was calculated as the coefficient of variation of seabed slope within a particular sea area. We also compared the Spearman rank correlation coefficients between known diversity (total species richness, alien species, and endemics) and available resources: numbers of taxonomic guides and experts.

The NRIC summarised their research resources, state of knowledge of taxa, and taxonomic expertise. Some also distinguished how many species were endemic, an indicator of how unique their biota was and enumerated alien species, an indicator of human-mediated disturbance to their ecosystems. The state of knowledge of each taxonomic group was classified from 1 to 5 (5 =  very well known: >80% described, identification guides <20 years old, and current taxonomic expertise; 4 =  well-known: >70% described, identification guides <50 years old, some taxonomic expertise; 3 =  poorly known: <50% species described, identification guides old or incomplete, no present expertise within region; 2 =  very poorly known, only few species recorded, no identification guides, no expertise; 1 =  unknown, no species recorded, no identification guides, no expertise.

All NRIC reported what they considered the main threats to marine biodiversity in their region, citing published data and expert opinions. Although their reports were not standardised, we grouped the threats identified into several overarching issues. We integrated these data on biodiversity threats so as to rank each threat from 1 (very low) to 5 (very high threat) in each region.

## Results

### Species richness

**Figure 2:**
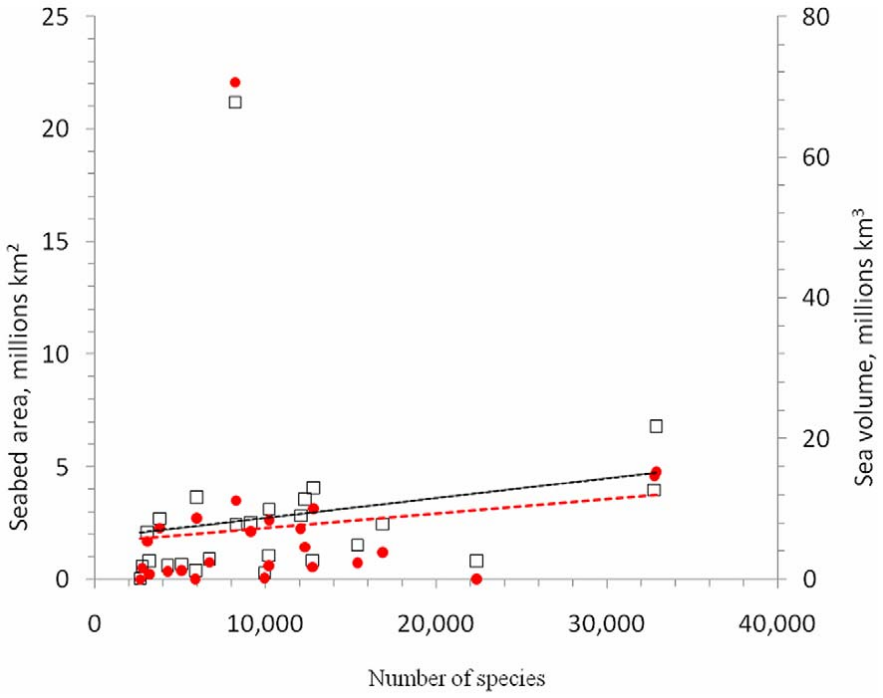
The relationship between total number of recorded species in each region to sea volume (solid red dots, dashed line, millions km^3^), and seabed area (squares, solid line, millions of km^2^) with linear trend lines shown.

The NRIC regions with most recorded species were Australia and Japan, each reporting over 32,000 species, and China, which had over 22,000 species (Table 1). However, most species per unit area were reported for South Korea, China, South Africa, Baltic Sea, and Gulf of Mexico. In contrast, Alaska, Arctic, Antarctica, and Patagonian Shelf have 10 times fewer species per area. While there were generally more species per unit seabed area and sea volume, the correlation was weak (r_s_ = 0.5) but significant (P<0.05) for area only (Figure 2, Table 2). Exclusion of the Southern Ocean, Antartica, which could be considered an outlier, increased the correlations and both area and volume became significant.

**Table 2:** The Spearman rank correlation coefficients between the metrics of diversity (number of all, alien and endemic species), state of knowledge index, resources (species identification guides, taxonomic experts), and NRIC size (area, volume, topographic variation) analysed in this paper.

	Number of species	Aliens	Endemics	Knowledge	Guides	Experts	Seabed area
Aliens	0.43						
Endemics	0.00	0.11					
Knowledge	**0.82** ***	**0.64** ***	0.10				
Guides	**0.70** ***	0.30	*−0.71* *	**0.72** ***			
Experts	0.34	0.28	*−0.69* *	0.39	0.43		
Seabed area	**0.50** ** (0.55***)	0.43	0.55	0.37	0.35	0.19	
Volume	0.37 (0.41**)	0.27	0.43	0.19	0.19	0.04	**0.94** ***

*P<0.07 in italics;

**P<0.05 bold,

***P<0.01 bold and underlined. Figures in parentheses represent correlations following exclusion of the Southern Ocean (Antarctica).

In almost all regions, three major taxa—Crustacea, Mollusca, and Pisces—together contributed about half of all species richness, while Protozoa and algae contributed 10% each (Table 3). The proportion that each taxon contributed to the regional species richness varied considerably, as some taxa contributed more than double or less than half the mean and median levels. The Crustacea contributed 22%–35% of species for Alaska, Antarctica, Arctic, Brazil, California, Caribbean, Eastern Canada, and Humboldt regions, but only 10% for the Baltic. Mollusca contributed 26% of the species in Australia and Japan, but only 5%–7% of the species in the Baltic, California, Arctic and eastern and western Canada. Fish contributed 18%–32% of species for the southeast and northeast USA, Tropical Eastern Pacific, and Tropical Western Atlantic, but only 3%–6% for the Arctic, Antarctica, Baltic, and Mediterranean. The “plants and algae” (largely algae) contributed 28%–38% of the species in the Baltic, Arctic, Atlantic Europe, and Western Canada, but only 5% in Antarctica, Caribbean, China, Humboldt, Tropical Eastern Pacific, and Tropical Western Atlantic. Of the less species rich taxa, Annelida (mostly polychaete worms) contributed 28% of the species for the Tropical Eastern Pacific, but only 3% for Japan. The taxa with the most variable proportions were the “plants and algae,” “other invertebrates,” and “other vertebrates”; reflecting variation in their classification between regions. In contrast, the Crustacea and Mollusca, clearly distinguished taxa, showed the least variation in their proportions across the regions.

**Table 3:** The percent of species per taxon in the geographic regions listed in Table 1, including the mean, median, coefficient of variation (CV  =  SD/mean), and percent of regions in which a taxon contributed over 10% of the species in each region.

	Total Eukaryota	Crustacea	Mollusca	Pisces	Protozoa	Plants and algae	Annelida	Cnidaria	Other invertebrates	Platyhelminthes	Echinodermata	Porifera	Bryozoa	Other vertebrates	Tunicata	Difference from mean SD
% areas >10%		81	58	58	35	29	23	0	13	3	0	0	0	0	0	
Australia	32,889	*19*	**26**	*16*	2	6	5	5	3	2	5	5	3	1	3	0.08
Japan	32,777	*19*	**26**	*12*	*14*	7	3	6	4	1	3	2	1	0	1	*0.08*
China	22,365	*19*	*18*	*14*	**21**	5	5	6	2	2	3	1	3	1	1	0.07
Mediterranean	16,848	*13*	*13*	4	**24**	7	7	4	*13*	6	1	4	2	0	1	0.07
Gulf of Mexico	15,374	*17*	*16*	*10*	*14*	*13*	6	5	4	5	3	2	2	3	1	0.06
New Zealand	12,780	*17*	*18*	*10*	*12*	*11*	4	6	4	2	4	4	5	1	1	0.06
South Africa	12,715	*18*	**24**	*15*	2	7	6	7	5	3	3	3	2	2	2	0.07
Atlantic Europe	12,270	*18*	*11*	9	4	**28**	*13*	4	0	2	2	4	3	2	1	*0.08*
Caribbean	12,046	**24**	**25**	*11*	7	5	5	8	3	1	4	4	1	0	1	*0.08*
Humboldt Current	10,186	**31**	*12*	*11*	7	5	6	5	8	2	4	2	4	2	1	0.08
USA California	10,160	**26**	7	9	9	9	8	4	7	*14*	3	1	1	1	1	0.07
Korea	9,900	*14*	*19*	*11*	3	9	5	3	**25**	1	2	3	1	2	1	0.07
Brazil	9,101	**22**	**20**	*14*	3	9	*11*	6	3	0	3	4	1	2	1	0.07
Hawaii	8,244	*16*	*16*	*15*	*10*	*12*	4	6	3	8	4	2	2	1	1	0.06
Antarctica	8,200	**35**	9	4	8	4	7	6	7	2	7	3	4	3	1	*0.08*
SA Trop East Pacific	6,696	*13*	*13*	*18*	*14*	5	**28**	2	1	0	3	1	1	1	0	*0.09*
Alaska	5,925	**26**	8	7	*13*	7	9	4	*10*	2	3	3	6	2	1	0.06
Baltic	5,865	*10*	5	3	**20**	**30**	7	2	*13*	5	1	0	1	2	0	*0.09*
USA NE	5,045	*16*	*17*	*19*	1	*12*	*14*	4	3	2	3	1	3	4	1	0.07
USA SE	4,229	*16*	*17*	**28**	4	8	9	9	1	0	0	3	2	2	1	*0.08*
Patagonian Shelf	3,776	*16*	**22**	*14*	0	7	5	7	5	1	5	7	4	5	1	0.06
Canada Eastern	3,160	**23**	7	*17*	*19*	*12*	*14*	3	2	0	2	0	0	1	0	*0.08*
Canada Arctic	3,038	**24**	5	6	*12*	**36**	*11*	2	2	0	1	0	0	1	0	**0.11**
SA Trop West Atlantic	2,743	*19*	*16*	**32**	2	5	6	5	2	0	4	1	0	8	1	**0.09**
Canada Western	2,636	*18*	7	*14*	4	**38**	*14*	0	2	0	1	0	0	1	0	**0.11**
Mean	10,759	*19*	*17*	*12*	*10*	*10*	7	5	5	3	3	3	2	2	1	0.06
CV		−0.29	−0.38	−0.54	−0.67	−1.00	−0.74	−0.39	−1.02	−0.97	−0.49	−0.65	−0.65	−1.04	−0.51	

Taxa that contributed >10% are indicated in italics, and >20% in bold. Taxa are sorted from most to least average richness, and regions from most to least total species richness. SD  =  Standard deviation.

Australia and New Zealand recorded over 9,000 and 6,500 endemic species respectively, while Antarctica and South Africa each recorded over 3,500; and the Caribbean, China, Japan, and Mediterranean had less than 2,000 each, and the Baltic only 1 endemic species (Table 4). The number of endemic species was positively correlated with species richness, region area and volume, and state of knowledge (Table 2). Although these correlations were only significant at P<0.07, it should be noted that only eight NRIC provided estimates of endemism. Because Australia did not provide estimates for all taxa, its endemism of 28% is underestimated and may be closer to the 45% for Antarctica or 51% for New Zealand. In contrast, the number of endemic species was negatively correlated with the number of identification guides and experts (P<0.07, Table 2).

**Table 4:** The number of endemic plants, invertebrates, and vertebrates reported for NRIC regions.

NRIC region	Plants	Invertebrates	Fish	Other vertebrates	Total	Number of species	% endemics
Antarctica	—	—	—	—	3,700	8,200	45
Australia	—	7987	1298	—	9,286	32,889	28
Baltic	1	0	0	0	1	5,865	2
Caribbean	—	868	704	1	1,573	12,046	13
China	142	1387	70	2	1,601	22,365	7
Japan	—	1508	364	0	1,872	32,777	6
Mediterranean	171	844	80	3	1,098	16,845	7
New Zealand	225	6014	278	43	6,560	12,780	51
South Africa	—	3269	280	—	3,549	12,715	28
Total	538	21,639	3,074	49	25,300	150,617	17

### State of knowledge

**Figure 3:**
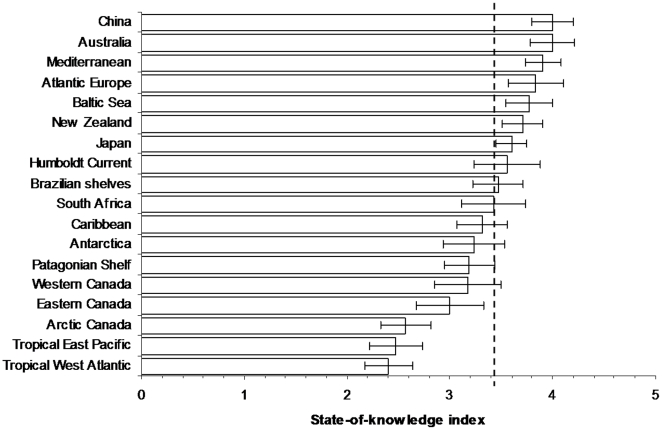
The regions ranked by their state-of-knowledge index (mean ± standard error) across taxa. Dashed line represents the overall mean.

The state-of-knowledge index had a mean value of 3.6±0.9 (mean ± standard error) over all regions (n = 18) (Figure 3), and was significantly correlated with species richness (Table 2). This indicated that most taxonomic groups were poorly known (<50% species described, identification guides old or incomplete, no present expertise within region) or well known (>70% described, identification guides <50 years old, some taxonomic expertise), depending on the group. Australia, China, and all three European regions, showed the highest values of knowledge by taxonomic group over the mean, while the Tropical West Atlantic, Tropical East Pacific and Canadian Arctic were well below it (Figure 3). Deep-sea areas in the Mediterranean Sea, Japanese waters, Southern and Indian oceans, South African, Canadian and U.S. waters, Australia, the Caribbean and South America (with the exception of the Brazilian shelf) were highlighted in regional revisions as more poorly known than coastal environments, and this is probably the case everywhere because of the practical difficulties in sampling deeper waters. Other regions identified as less investigated were coral reefs, ocean trenches, ice-bound waters, methane seeps, and hydrothermal vents in the Asian-Pacific region [9]; the southern and eastern Mediterranean Sea [8]; estuaries, coastal areas and coral reefs of the Indian Ocean [25]; and many habitats such as intertidal rocky shores in Canadian waters [23] and large regions of Southern America and the Indian Ocean [10], [25]. These studies also highlighted that their data had a limited spatial and temporal resolution.

**Figure 4:**
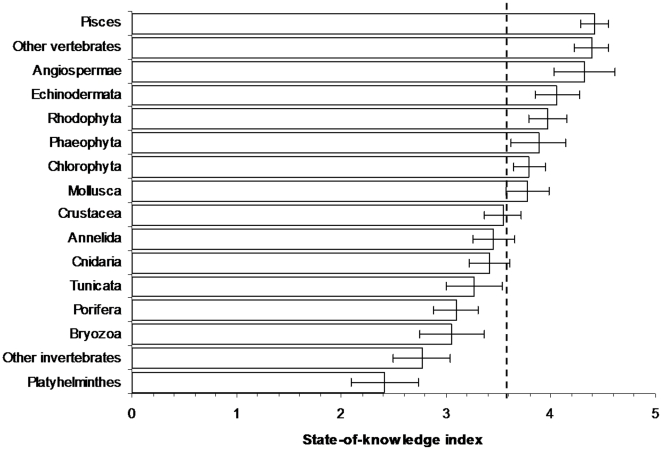
The taxonomic groups ranked by their state-of-knowledge index (mean ± standard error) across regions. Dashed line represents the overall mean.

Across taxa, the state-of-knowledge index had a mean value of 3.9±0.1. Taxa with a score over 4 were Pisces (fish) and other vertebrates, Angiospermae (flowering plants), Rhodophyta (red algae), Phaeophyta (brown algae), and Echinodermata (starfish, urchins); scores of less than 4 were recorded for other invertebrates (Figure 4). Platyhelminthes (flat worms), Bryozoa (sea mats), Porifera (sponges), Tunicata (sea squirts), and Cnidaria (corals, hydroids, jellyfish) ranked under the mean (Figure 4). Several regions specifically reported that less well studied taxa were: several eukaryotes and many forms of prokaryotes in the New Zealand EEZ; cryptic groups in Australia; bacteria, cyanophyceae, diatoms (Chrysophyta) and meiobenthos in the Caribbean; microorganisms, meiobenthos and parasites in the Baltic Sea; small body size taxa in South Africa, the Mediterranean, Canada, and United States; while nematodes, foraminiferans, and some macrofauna and megafauna remained largely unknown in the deep Mediterranean Sea [28]. In the Southern Ocean database, there were more distribution records for molluscs and echinoderms than for other invertebrates [16]. Even in areas that were highly ranked for mean knowledge by taxa, scientists were still discussing the total number of fish or other vertebrate groups, such as in the Mediterranean Sea [8].

Apart from China [24], Europe [33], and New Zealand [34], most regions lacked recent authoritative inventories of their species. This complicated estimation of the number of species in those regions because of the diverse literature and the need to account for synonyms. Estimating the number of undescribed species was difficult. However, undescribed species were estimated at 39–58% of the regional total for Antarctica, 38% for South Africa, 70% for Japan, 75% for the Mediterranean deep-sea, and 80% for Australia. New Zealand had 4,111 undescribed species in its specimen collections, which would comprise 25% of the known species, but clearly is a minimum estimate because many species will not yet have been collected and distinguished in collections.

### Resources: guides, experts, and facilities

**Figure 5:**
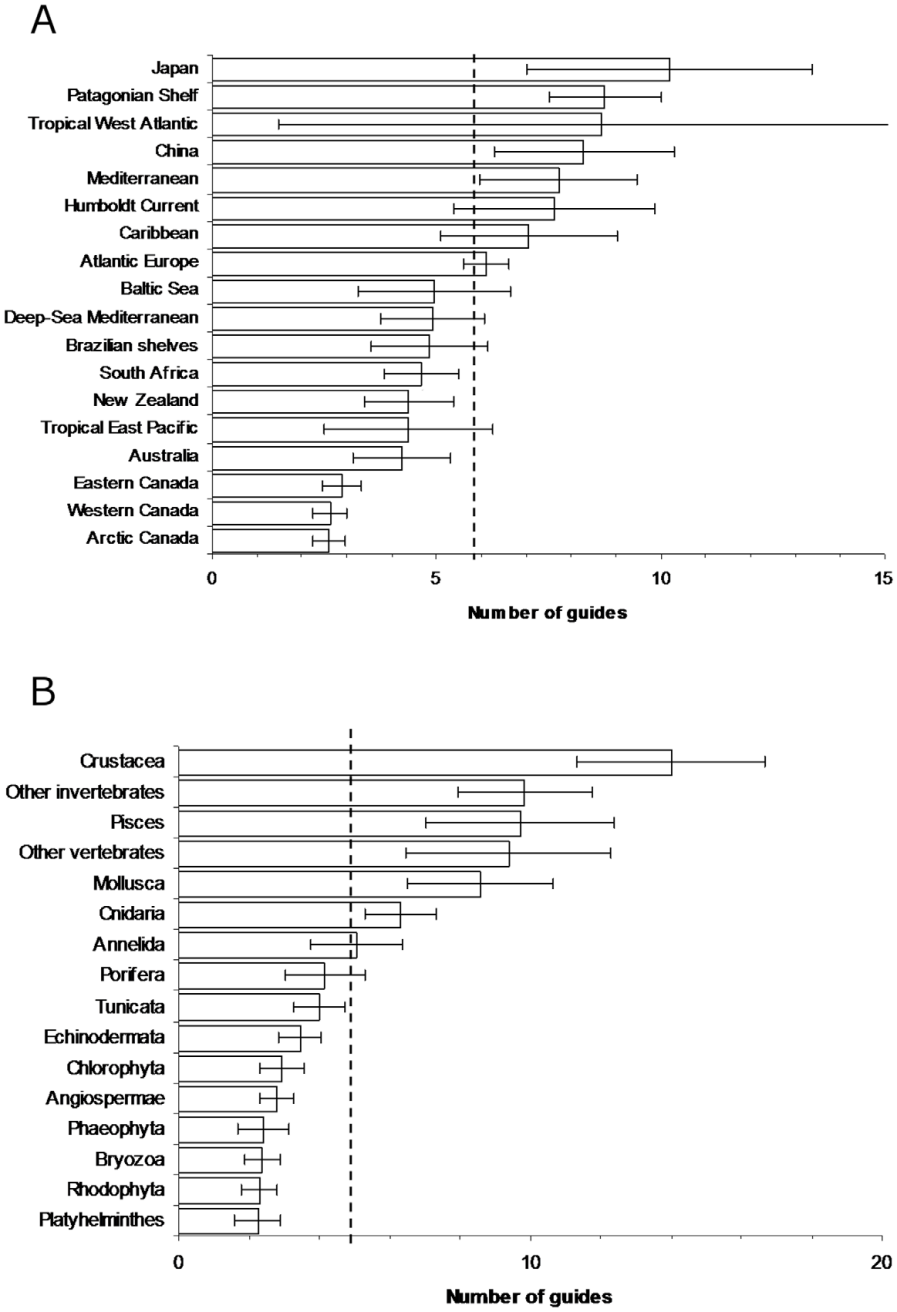
The mean (± standard error) number of species identification guides across (a) major taxonomic groups for each region, and (b) across regions for each taxon. Dashed line represents the overall mean.

**Figure 6:**
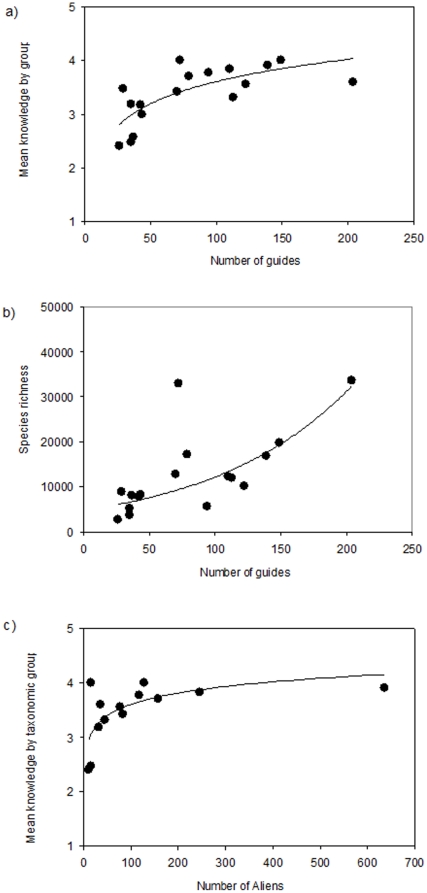
Relationship between the number of identification guides and (a) mean knowledge by group and (b) total species richness, and (c) the relationship between knowledge by taxonomic group and number of alien species in the NRIC regions.

We found that the main taxonomic groups had on average 6.0±0.7 species identification guides per region (Figure 5a), but that these resources varied from very few for Bryozoa and Platyhelminthes to 14 guides per region for Crustacea (Figure 5b) and up to 20 guides for a given group. Higher numbers of guides for major taxa were reported in Japan, and lower numbers were reported in Australia, New Zealand, Tropical Eastern Pacific, South Africa, and Canada. Resources also varied notably between taxonomic groups, with more guides for Cnidaria, Mollusca, Crustacea, and Pisces. The number of guides was significantly and positively correlated with the state of knowledge and species richness (P<0.01) (Table 2) (Figure 6a, b).

**Figure 7:**
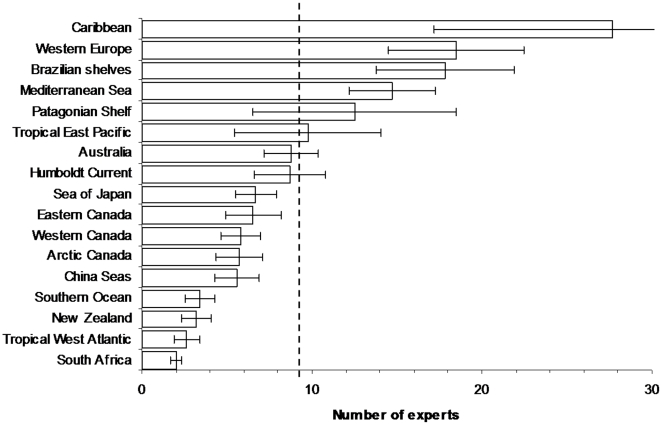
The number of taxonomic experts per taxon for each region (mean ± standard error). Dashed line represents the overall mean.

There were on average 9.4±1.7 experts per taxonomic group in each region (Figure 7). The Caribbean, Atlantic Europe, Mediterranean Sea, and Brazilian shelves showed the highest number of experts, while South Africa and the Tropical West Atlantic ranked the lowest. The number of taxonomic experts was not significantly correlated with species richness, species identification guides, or NRIC size (Table 2).

Almost all countries with a coastline had one or more marine biodiversity-related research facilities. However, the number of field stations per country was highly variable from one or only a few in the developing world, to several tens and even more than 100 laboratories in Europe, the United States, and Antarctica. The availability of research vessels (RV, ships) is another indicator of a country's investment in exploring its offshore marine environment. This research infrastructure was unevenly distributed globally. While the United States had hundreds of boats and research vessels (including 41 vessels over 40 m long), and Japan had more than 25 large vessels (over 500 tons gross), most other countries or regions around the world had few to none.

### Threats to diversity

The NRIC reported overfishing, habitat loss, and pollution (contamination by xenobiotics and eutrophication), to be the greatest threats to biodiversity in the regions, followed by alien species and impacts of warming due to climate change (Table 5, Text S1). While eutrophication has been the best-known cause of hypoxia, several reviews noted how climate change may also contribute to more hypoxic conditions. The more enclosed seas—Mediterranean, Gulf of Mexico, China's shelves, Baltic, and Caribbean—were reported to have the most threatened biodiversity at a global scale because of the cumulative impact of different variables. Other impacts reported less frequently, and so not summarised in Table 5, were related to aquaculture and maritime traffic, which were considered especially important in the Mediterranean Sea [8].

**Table 5:** Summary of the major threats to marine biodiversity in different areas reported by the regions (Table 1).

	Overfishing	Habitat loss	Pollution	Alien species	Temperature	Hypoxia	Acidification	Total	Median
Mediterranean	5	5	4	5	5	2	1	27	5.0
Gulf of Mexico	5	5	5	4	2	3	1	25	4.0
China	5	5	5	2	2	3	1	23	3.0
Baltic	4	3	4	3	4	3	1	22	3.0
Caribbean	4	4	4	4	2	2	2	22	4.0
USA Southeast	4	4	3	3	3	2	3	22	3.0
Brazil and Tropical West Atlantic	4	4	3	3	3	3	2	22	3.0
Humboldt Current and Patagonian Shelf	4	3	3	3	2	4	2	21	3.0
North Indian Ocean	3	4	4	3	3	2	2	21	3.0
Tropical East Pacific	3	3	3	3	3	3	2	20	3.0
South Africa	3	2	4	4	2	4	1	20	3.0
New Zealand	4	3	2	4	2	1	3	19	3.0
Atlantic Europe	4	2	4	2	4	1	2	19	2.0
USA Northeast	4	3	3	2	3	2	1	18	3.0
Japan	3	3	3	2	3	1	2	17	3.0
Canada (all)	2	4	2	2	5	0	1	16	2.0
Australia	3	3	2	3	2	0	1	14	2.0
Antarctica	2	2	2	0	1	0	2	9	2.0
Total	66	62	60	52	51	36	30		
Median	4.0	3.0	3.0	3.0	3.0	2.0	2.0		

Each threat was scored from 1 to 5 (minimum to maximum) across a comparative scale among different regions. Some regions (e.g., Australia) reported only known threats rather than predicted threats. Table is sorted by reported greatest threats and areas with greatest impacts. Median values of each threat and for each region are also reported.

Of the reported regional estimates for the number of alien species, the Mediterranean estimates of more than 600, or 4% of the species, was by far the highest (Table 6). This number may be as high as 1,000 species if unicellular aliens and foraminiferans are included [35], [36]. A high number of alien species was also reported for Atlantic Europe and the Baltic Sea (2% of the biota), New Zealand, and Australia. Lower numbers of alien species were recorded from China, and the Tropical East Pacific and Tropical West Atlantic coasts of South America. On average, there were 122±15 aliens per NRIC region. By taxonomic groups, molluscs, crustaceans, and fish contributed most alien species. The number of alien species was not correlated with the total richness, but was correlated with the state of knowledge (Table 2) (Figure 6c).

**Table 6:** The number of alien species reported for each region by taxonomic group.

	Mediterranean	Atlantic Europe	New Zealand	Australia	Baltic	South Africa	Humboldt Current	Caribbean	Japan	Patagonian Shelf	China	Tropical East Pacific	Tropical West Atlantic	Number of occurrences	Mean
Mollusca	200	55	12	22	12	11	7	6	11	3	3	2	3	13	26.7
Crustacea	106	61	17	10	33	21	4	7	10	9	7	0	1	12	22.0
Pisces	116	39	3	12	29	1	35	15	1	1	0	10	2	12	20.3
Annelida	75	15	21	20	12	7	8	2	10	4	0	1	1	12	13.5
Rhodophyta	73	25	12	10	4	3	10	3	0	3	1	0	3	11	11.3
Cnidaria	3	15	23	10	5	13	1	5	1	1	0	0	0	10	5.9
Bryozoa	1	0	24	24	1	6	2	2	0	5	0	0	0	8	5.0
Tunicata	15	9	11	2	1	9	5	1	2	6	2	1	0	12	4.9
Phaeophyta & Chromista	23	5	10	6	7	0	1	0	0	1	1	0	0	8	4.2
Chlorophyta	17	5	0	2	2	1	1	2	1	0	0	0	0	8	2.4
Porifera	0	0	17	4	0	1	2	1	0	0	1	0	0	6	2.0
Dinoflagellata	0	10	0	2	2	3	0	0	0	0	0	0	0	4	1.3
Platyhelminthes	0	6	2	1	2	0	0	0	0	0	0	0	0	4	0.8
Echinodermata	5	0	0	3	0	2	0	0	0	0	0	1	0	4	0.8
Other invertebrates	2	0	2	0	3	3	0	0	0	0	1	0	0	5	0.8
Angiospermae	1	0	0	0	1	2	1	1	0	0	0	0	0	5	0.5
Other vertebrates	0	0	0	0	3	0	0	0	0	0	0	0	1	2	0.3
Foraminifera	0	0	3	0	0	0	0	0	0	0	0	0	0	1	0.2
Total aliens in region	637	245	157	128	117	83	77	45	36	33	16	15	11	13	122.2
% all species alien	4	2	1	<1	2	1	1	<1	<1	1	<1	<1	<1		1

## Discussion

### Species diversity

The total number of marine species in the NRIC regions, and globally, is still uncertain because so many species remain to be sampled, distinguished, and described. An estimated 25%–80% of species remained to be described in Australia, Japan, Mediteranean deep-sea, New Zealand, and South Africa, also regions of high species richness. We may expect the proportion of undescribed species to be toward the higher end of this range for the tropics of Asia and the Pacific. Thus, the proportion of undiscovered species may be close to 70%–80% of all marine species. The current estimate of described species is 230,000 [1], suggesting there may be 1 million to 1.4 million marine species living on Earth.

In most regions, Crustacea, Mollusca, and Pisces were the most species-rich taxa. The proportion of taxa in well-known regions, such as Europe, has been used to estimate how many species of other taxa may occur in less well studied areas (e.g. [1], [37]). However, whether these proportions, even at higher taxonomic levels such as phylum and class, are constant worldwide has not been demonstrated [4]. That the mean and median proportions of species richness across taxa in the NRIC regions are within 2% of each other (Table 3) may suggest that the average across regions is representative of a global pattern. Indeed, it may represent a global average which may be useful for some purposes. However, there was great variation between regions in the relative species richness of well-known taxa such as fish (3%–32%) and clearly classified taxa such as Crustacea (10%–35%) and Mollusca (5%–26%).

The high proportions of other taxa in some regions may reflect either a different classification of species or errors, which could account for the proportions of the “other” taxa categories being more variable than distinctly named taxa. Similarly, the high proportion of Angiospermae in western Canada may reflect inclusion of salt-marsh plants excluded from other inventories. Until species-level inventories compiled using a standardised classification at species level are compared, it will not be possible to conclude whether these higher taxa have the same proportions across the world's oceans. Even then, variation in taxonomic effort with regions will affect the relative number of species between taxa, as indicated by the general decrease in the state-of-knowledge index with increased variation in proportions of taxa across regions. Indeed, Griffiths [37] reported how uneven taxonomic effort explained the apparently low richness of some taxa in southern Africa. In the present study, the low proportion of annelid worms recorded for Japan seems unlikely to be true and probably reflects a need for greater taxonomic effort.

The variation in the richness of the more species-rich and well-known taxa, such as fish, suggests that the proportions that taxa contribute to regional diversity are not comparable around the world. For the relative species richness to be the same throughout the world's oceans would require similar patterns of dispersal, speciation, and extinction geographically. This seems unlikely as the diversity of taxa tends to vary with environment. For example, reef-building corals are most diverse in the tropics and annelid worms in sediments, and echinoderms are scare in estuaries. Further evidence is thus required to support the use of taxonomic ratios in biogeography.

### Sampling effort

The poor or moderate correlations between species richness and the size of NRIC regions were surprising considering the well-established species-area relationships (e.g. [38]). This may indicate that the species-area relationship does not hold for the oceans, or (more likely) reflects a lack of sampling in large areas within regions or variable taxonomic effort. Indeed, the state of taxonomic knowledge was only considered well known for Australia, Atlantic Europe, China, and the Mediterranean regions. European seas are probably the best studied globally [2], while Australia, Japan, and New Zealand may be the best studied within Australasia and the western Pacific.

Comprehensive identification guides for the many less well studied invertebrates are often unavailable, so these species are studied only by specialists. Thus, the lack of specialists within regions will result in apparently fewer species in these groups. Furthermore, a range of habitats were insufficiently studied in the regions, particularly deeper seas. As the areal extent of such habitats varies between regions, this would contribute to the poor species-area relationships that we found. Even within well-studied NRIC regions, there were differences between subareas (e.g., Mediterranean Sea [8]), and NRIC varied in the range of climatic regions they included. For example, Australia ranged from tropical to sub-Antarctic.

The large number of endemic species reported from New Zealand (51%), Antarctica (45%), Australia (28%), and South Africa (28%), was remarkable. Similarly, a contemporary analysis found that most endemic marine fish genera occurred in southern Australia (50 genera), southern Africa (36), Mediterranean (5), and the Red Sea (4) and that 24% of Australian fish species were endemic and that New Zealand and the Pacific islands were rich (15%–20%) in endemic species [4]. All three areas reported in the present study (Australia, New Zealand, South Africa) are relatively isolated, with ancient Gondwanan origins. They may have suffered fewer extinctions from climate cooling (e.g., glaciation), or they may have been more easily recolonised from regions unaffected by climate cooling [39]. We found that the number of endemic species and the number of identification guides and taxonomic expertise were strongly negatively correlated (r_s_ = −0.71, −0.69). This suggested that further study reduced the number of species considered endemic. In the Mediterranean Sea, for example, the level of endemism has decreased recently as more information became available from adjacent areas [8]. Thus, whether more data from adjacent regions, such as middle Africa, and the Indo-Pacific islands will reduce the proportion of endemics in the above NRIC regions remains to be seen.

### Threats to biodiversity

Over-fishing was reported to be the greatest threat to marine biodiversity in all regions (Table 5, Text S1). Habitat loss posed a similar level of threat in several regions, while pollution ranked as the third-greatest threat overall. The fact that these threats were reported in all regions indicates their global nature. Examples of overfishing occurred throughout the NRIC regions and across the range of taxa harvested. These not only deplete the exploited fish stocks themselves but deplete bycatch species abundance (e.g., turtles, albatrosses, mammals), and have consequent indirect impacts on ecosystems through altered food webs. Marine habitats are being lost as a result of coastal urbanisation, sediment runoff from land, eutrophication and hypoxia due to land-derived nutrients (e.g., sewage, agricultural fertilizer), sea level rise, melting of polar ice sheets, dynamite fishing, fishery bottom trawling and dredging, aggregate dredging and extraction, and trophic cascades leading to a benthos dominated by sea urchins and lacking in seaweed cover. In addition to nutrient pollution (eutrophication) and associated hypoxic events called “dead zones”, there are more toxic contaminants, such as oil pollution. While efforts are under way to reduce discharges of persistent contaminants (e.g., PCBs, mercury), they continue to occur in long-lived marine vertebrates. The reduction in use of the highly toxic antifoulant agent tribuytltin (TBT) should lead to a recovery of gastropod and bivalve populations near harbours (e.g. [40]). Large areas of garbage collecting in ocean gyres have been discovered, as well as littering of the seabed and entangling of marine species (e.g. [41], [42]). “Climate change” encompasses a range of environmental threats that vary geographically. They include temperature change, ocean acidification, sea-level rise, and consequent changes to ocean stratification, upwellings, currents, and weather patterns. Biodiversity is already responding to some of these changes (e.g. [43], [44], [45]), and how it will change in the future is difficult to predict because of the complexity of biodiversity, from genes to species to ecosystems.

### Knowledge and resources

We suggest that the significant correlations between the number of species identification guides and species known to occur within regions indicate that it is easier to discover species when good identification guides are available. Thus, the production of regularly updated and comprehensive guides to all species in regions should be a priority for both research and environmental management (e.g., detection of invasive species, rare species, and pests). However, apart from guides with a commercial market (e.g., birds, mammals, fish), there are few incentives to publish comprehensive species identification guides in comparison to short papers in science journals. Most guides are published as books that do not receive citation-based “Impact Factors” as do papers in journals, and thus do not similarly add to the citation record of scientists. The decline of the past practice of citing the guides used to identify species in ecological and other studies has further reduced the apparent impact of authors' work [46]. Several changes of practice are needed to address this issue: (a) scientists should cite the references used to confirm the identification of species in their papers, (b) authors should publish guides in open-access, online resources where citations can be tracked and recorded, and (c) publishers and employers should encourage both of these practices. The production of such guides may be the most valuable service taxonomists can provide to science and society, but this requires considerable effort in describing new species, better describing known species, and resolving taxonomic issues and nomenclature that are often not obvious to the user of a guide. However, the availability of guides opens a field of study to many more people, including professionals, students, and amateurs and will thus help in the discovery of species new to science and in advancing the knowledge of regional biodiversities.

The lack of a clear species-area relationship across the regions was indicative of the lack of sampling in major areas and habitats of the oceans, and insufficient species identification guides and taxonomic expertise. The more developed countries had more marine research laboratories and ships. However, they also suffered from insufficient knowledge for many taxonomic groups and declining taxonomic expertise [5], [23], [25]. That the number of experts did not correlate with any metrics of diversity, resources, or knowledge (except the number of endemic species) may indicate the variable distribution of expertise globally and even within a region, but may also have been influenced by the difficulty of defining who is an expert. Most undiscovered species are likely to be found in the tropics, deep seas, and seas of the Southern Hemisphere, including many developing countries. It is unlikely that every country needs expertise in every taxonomic group or large research facilities, so collaboration between countries, as already occurs informally, is critical to developing knowledge on all species. There is potential for further benefits, cost-efficiencies, and quality control in taxonomy, ecology, and resource management through collaboration between countries and international organisations. There appear to be roles here for organisations such as the Intergovernmental Oceanographic Commission of UNESCO and the Global Biodiversity Information Facility (GBIF) to coordinate cooperation between countries (reflecting their national memberships); the International Association for Biological Oceanography as part of the International Union of Biological Sciences and thus the International Council of Scientific Unions, which represent the national academies; and grass-roots taxonomic societies involved in networking through conferences and online databases (e.g., the Society for the Management of Electronic Biodiversity Databases, Crustacean Society).

The online publication of existing and new marine biodiversity data is now possible, as demonstrated for species distribution data by the Ocean Biogeographic Information System and GBIF, and for taxonomic data by the World Register of Marine Species [47], [48]. Such integrated, open-access, online data publication needs to expand to include ecological and other data, and it requires regular updating [46]. Online publication is most likely to succeed if mechanisms for citation are both implemented by the online publishers and used by researchers [46] and if scientists publish in such open-access media.

### Future needs

To meet the future needs and challenges in studying marine biodiversity, we recommend improved coordination between institutions, including museums, fisheries institutes, government and intergovernmental agencies, and universities at the international, national, and regional levels to (1) formally agree on key gaps in knowledge, (2) appoint staff to fill gaps strategically as positions become available, (3) facilitate staff exchange to fill gaps and train staff in other countries, (4) facilitate graduate training to address gaps, and specifically to cope with the progressive loss of taxonomic expertise, (5) host workshops (including field studies) and symposia to generate team-building and a sense of urgency and momentum amongst participants to address gaps, (6) support low-cost, open-access publication of knowledge through e-journals and authoritative online species information systems (including digital species identification guides), (7) develop new technologies for ocean exploration, knowledge discovery, data management and dissemination of results, and (8) encourage international collaboration between countries to facilitate field work, strategically build specimen collections, and publish data and knowledge online. Leadership for such coordination will need to come from champions in the scientific community, key institutions (e.g., those that host databases and publications), and countries that fund the institutions and scientists. This study comes at the end of a decade of the Census of Marine Life. We show that there remain major gaps in basic knowledge of marine biodiversity, taxonomically and geographically. Science and society would thus benefit from another decade of discovery that strategically builds on our findings.

## Supporting Information

Text S1 A more detailed review of the threats to marine biodiversity identified by the Census of Marine Life National and Regional Committees in their papers.
(0.16 MB DOC)
