# Supplementary material for: A Census of Marine Biodiversity Knowledge, Resources, and Future Challenges
Source: PLoS One. 2010 Aug 2;5(8):e12110. doi: 10.1371/journal.pone.0012110 (PMC2914025; doi:10.1371/journal.pone.0012110)
Supplement: Text S1 — A more detailed review of the threats to marine biodiversity identified by the Census of Marine Life National and Regional Committees in their papers. (0.16 MB DOC) [file pone.0012110.s001.doc]

**Text S1. A more detailed review of the threats to marine biodiversity identified by the Census of Marine Life National and Regional Committees in their papers.**

**Box 1. Examples of threats to marine biodiversity from fisheries.**

The impact of fisheries on biodiversity of the northeast and southeast United States has included severe reductions in upper trophic level predators (such as sharks, snappers, groupers, and Carangidae) and cascading responses through lower parts of the food web [1, 2, 3]. Populations of pelagic sharks, tunas, and mackerels (Scombridae) are also currently or periodically overfished. Marine mammals such as the Atlantic right whale and the Florida manatee are endangered [4]. Overfishing of individual species and fishing gear effects are likely to have an impact on the health of populations of associated algae, invertebrates, and other vertebrates.

Overfishing in the northern Gulf of Mexico has affected both commercial and recreational fisheries [5, 6]. In the southern Gulf of Mexico and the Caribbean, the shrimp fishery has almost completely ceased because of a combination of over-fishing, under-regulation, and environmental change [7]. Octopus, one of the most important fishery resources in this area (*Octopus maya* represents 80% of the catch, and *O. vulgaris* the rest), has been fully exploited [8], while conch and lobster fisheries on coral reefs have become commercially extinct through overharvesting [9]. Red grouper (*Epinephelus morio*) fishing on coral reefs has decreased since the 1970s. Heavy shark fishing led to reduced populations. Sales of shells, soft and hard corals, and hawks bill sea turtle carapaces continue in street markets of Veracruz, Mexico. The most endangered species include the Kemp’s Ridley sea turtle, the whooping crane, the piping plover, the reddish egret, and all the great whales [10, 11, 12].

Fisheries in California have caused the depletion of salmon and steelhead [13]. Seven species (widow rockfish, canary rockfish, yelloweye rockfish, dark blotched rockfish, bocaccio, Pacific ocean perch, and cowcod) currently have been overfished and are subject to rebuilding efforts. In the Georgia Basin, canary and yelloweye rockfish have been proposed for threatened status, and bocaccio for endangered status. In Japan, overfishing has caused the decline of wild fish catches and has driven an increase in aquaculture. Reef-forming corals of the Florida Keys are declining [4], and decades of fishing on aggregations of spawning reef fishes has resulted in declining abundance in aggregations, but no-fishing zones have been restoring populations of predatory fishes [14]. The effects of fishing on target species in Australia include effects on non-target species (e.g., turtle) that are being limited by the use of specific sampling devices [15, 16, 17, 18]. In New Zealand, overfishing, bycatch depletion, and diminishment of ecosystem services because of biodiversity loss have been documented. The exploitation of deep-water species of scampi (*Metanephrops challengeri*) and of selected demersal fish over an area of 2,400 km^2^ is negatively associated with invertebrate species richness and diversity.

Overexploitation of living resources in Antarctica, such as krill, fish, and their associated bycatch, is a major threat to the pelagic ecosystem. Benthic trawling in the South Georgia region was banned in the 1980s, but longline fishing continues. Commercial fishing in Antarctica is heavily regulated, but unlicensed vessels keep on fishing illegally. Historically, overexploitation first concerned whales and seals, but commercial sealing ended in the 1950s and all but scientific whaling ended in the mid-1980s.

In the Tropical East Pacific, fisheries not only threaten fish and benthic invertebrate species but also exert detrimental effects on sea turtles (*Lepidochelys olivacea* and *Chelonia agassizii* [19]). In the Patagonian Shelf, several fish and invertebrates, mainly molluscs and crustaceans, are largely exploited [20, 21]. Bycatch of seabirds, marine mammals, and turtles are relevant. Bottom trawling dominates coastal and deep-sea fishing and produces large amounts of discards of benthic invertebrates, accounting up to 80% of the catch [22]. Bycatch affects at least 4 species of marine turtles, some 20 species of birds, and 7 species of mammals (sea lions, elephant seals, and dolphins). In the Tropical West Atlantic, fishing activities on species with commercial value is particularly intense [23]. Endangered species include the catfish, scienids (*Micropogonias furnieri* and *Macrodon ancyclodon*), carangids (*Selene setapinnis*, *Trachinotus cayannensis, Trachinotus carolinus*), the grouper *Epinephelus flavolimbatus,* and the snapper *Rhombopolites aurorubens*. The most important species captured with lines are the “carite sierra” (*Scomberomorus cavalla*) and the barracuda (*Sphyraena barracuda*) [24]. Approximately 80% of the resources of Brazilian waters are currently overexploited.

Fisheries in the northeast Atlantic have a negative impact on benthic diversity, production, and community structure [25, 26], and large parts of the area are fished 5-10 times a year. In the Baltic, a remarkable increase in fishing mortality has become evident since the mid-1940s [27]. The impact of fishing on the Baltic fish stocks is very important [28]. In the Mediterranean Sea, the depletion of species is evident on historical time scales (e.g., in the Adriatic [29]). Fisheries are responsible for 93% of the observed depletions. Several fish resources, including macrophytes, sponges, cnidarians, echinoderms, molluscs, arthropods, polychaetes, ascidians, and other invertebrates, are highly exploited or overexploited [30-34]. Direct exploitation of coastal resources by South African fisheries increased dramatically in the 1950s [35-37] but subsequently declined. Overexploitation of pelagic and demersal stocks in the Indian Ocean is a recognized phenomenon [38] and is a serious threat in Chinese waters. The Chinese shrimp *Fenneropenaeus chinensis*, a large, endemic species with high economic value, is nearly extinct in Guangdong Province, the northern South China Sea. The Bohai-Yellow Sea population of Chinese shrimp has seriously declined and is an “endangered species.” The important fishery for large yellow croaker *Larimichthys crocea* in the East China Sea has collapsed and is also endangered; the stock size has decreased to only 71 t in the East China Sea shelf region. The horseshoe crab *Tachypleus tridentatus*, a major medicine resource primarily distributed in the northern South China Sea，has been seriously overfished and is now assessed as an endangered species. About 10% (26 over 256 species) of species of scleractinian corals are endangered, and the rest are “vulnerable endangered.” Of the Mollusca, 23 species are “endangered,” 22 are “critically endangered,” and 12 are “extinct.” Fifty-six species of Crustacea are now endangered, and of the two species of living fossil *Xiphosura*, one is endangered and the other is vulnerable endangered. Of the fish fauna, 270 species were endangered, 19 species critically endangered, and 4 are extinct. Also about one-third of the 150 species of the holothurid echinoderms are “endangered” because of overexploitation.

**Box 2. Examples of the threats to marine biodiversity from habitat loss.**

The effects of human encroachment and activity on coastal habitats reduces the amount of natural space, introduces pollutants, and impedes natural adjustments to the shore as sea level rises [29]. These effects are exacerbated by the impact of climate change on specific marine habitats. For instance, the destruction of natural habitats contributes to changes in the centers of distribution of several taxa, an effect that is particularly evident for species associated with the ice pack. A decline in reproductive success of mammals is being documented for species, such as ice seals, that use the ice cover to den and raise their pups on the ice [39]. Along the South African east coast, many shores consist of rock platforms bounded by sandy habitats above where rising sea levels may result in the loss of some upper intertidal species. Alteration in coastal habitats as a result of sea level rise, changing ocean water circulation, and upwelling patterns also is extending along the west coast of the United States. In the Gulf of Mexico, extensive coastal habitat damage and loss have been caused by intense hurricanes in recent years. Although habitat loss in the United States has been greatest in Louisiana, with the disappearance of 80-130 km^2^ of coastal wetlands each year, other states are experiencing significant loss due to coastal development and infrastructure in selected areas. In Texas and Louisiana, sea level rise has been compounded by the subsidence of some coastal lands as a result of water and petroleum extraction [40]. Also in the Florida Keys, coastal development continues to have an impact on habitats of estuarine species and estuarine-dependent stages of shelf species.

Caribbean coral reefs are already greatly degraded, declining in some instances from more than 50% live cover to less than 10% over the last two decades [41]. This decline is due to a combination of forces, including damage by hurricanes, sediment runoff, boat anchors, fish traps, grounded ships, dredging, collection, and dynamite fishing. High population pressure in coastal areas has also led to the conversion of many mangrove areas to other uses, including infrastructure, aquaculture, rice, and salt production, at a rate of about 1% per year since 1980; that is, about 413,000 ha of mangroves have been lost in the Caribbean in that period [42]. In many areas of the Caribbean and Tropical West Atlantic, mangroves and seagrass have been removed to “improve” bathing beaches and to allow access to shipping or to lay pipes and other submarine structures (e.g., [43]). In the Mediterranean Sea, the “cementification” of the coastal habitats is of increasing concern [44, 45].

In the Indian Ocean, mangrove deforestation is the most evident example of habitat loss [46]. In Malaysia and Vietnam, removal of mangroves has led to a phenomenal decline in their role in coastal protection and as nurseries for larval and juvenile forms [47]. In Indonesia, a decadal loss of mangrove cover to brackish-water shrimp farms comprised half a million ha in the early 1990s [48]. The loss of sand dunes and associated flora is nearly total in Goa (India). Coastal construction and the consequent loss of habitat are one of the major threats of biodiversity in the China Seas. Habitat loss and degradation are identified as the main impact on the diversity of 12 large taxonomic groups in the Mediterranean Sea. Coastal development, sediment loading, and pollution notably reduced the extension of important habitats for marine diversity such as seagrass meadows, oyster reefs, and beds of maerl and macroalgae. They have affected Mediterranean ecosystem functioning since well before the 1900s [49, 50]. Because most species depend strongly on their habitats (such as bryozoans, sponges, echinoderms, benthic decapods, and organisms of the suprabenthos and meiobenthos), their loss and degradation has a notable affect on marine diversity. But large, erect species of brown algae, as well as the Mediterranean seagrass *Posidonia oceanica*, are in decline because of environmental degradation mostly induced by human activities [31, 51]. Currently endangered Mediterranean species include marine mammals and sea turtles, which historically have been victims of exploitation but more recently have suffered the disruption of their nesting habitat and been killed as unwanted bycatch [52, 53]. Fishing activity in the Mediterranean is also causing structural and functional changes and ecosystem degradation (e.g., [54-57]). Similarly, the change, loss, or modification of habitat and the destruction by overfishing are threatening New Zealand coasts. Along the Patagonian Shelf, the degradation and disturbance of habitats and sediment resuspension are having an impact on benthic species, and several areas require urgent conservation measures, including specific actions to avoid or minimize the effects of the dredging nets. Bottom trawling is causing indirect impacts (physical destruction) on habitat structure [10, 58]. This is true also of the cold-water coral reefs of *Lophelia pertusa*, inhabiting the continental margins at temperate and high latitudes (e.g., Norwegian Sea, Arctic region, and northeast Atlantic), which are particularly susceptible to damage by trawling. The reefs can provide habitat for a species-rich epibenthos and fish community, which in many cases are not specifically associated with the coral itself but benefit from the available hard substrate for settlement and its complex three-dimensional structures [59-62].

**Box 3. Threats to marine biodiversity from contamination and eutrophication.**

The most significant threat from ships to the Arctic marine environment is the release of oil through accidental or illegal discharge. Oil can reduce insulating properties of marine mammals and seabirds, causing hypothermia, and can be fatal if ingested, inhaled, or absorbed. In the high-latitude habitats and in the northeast Atlantic, contamination activities related with the oil and gas industry pose an increasingly serious concern for the sustainability of ecosystem functioning and biodiversity. One study suggested that the decline of seal populations by about one order of magnitude was due to overhunting followed by toxic pollution [63].

Contamination and eutrophication have become problems in most of the densely populated and industrialized regions of the world [64,65]. Contamination and the degradation of water quality are becoming critical concerns in the Gulf of Mexico and stimulated the Governors’ Action Plan for Healthy and Resilient Coasts [66]. Increasing population densities and the expansion of agricultural and industrial activities are threatening the Caribbean region. The coastal zone of the Tropical East Pacific is increasingly contaminated (including Ecuador and the Galapagos Islands; [67]), which threatens the biodiversity of the region [68]. In the Tropical West Atlantic, contamination is caused by urban and agricultural development, dredging and flow navigation, runoff from the Orinoco and Amazon basins, oil and gas exploitation, port activities, and maritime transit [69]. Hydrocarbon spills, heavy metal contamination, and their biological effects cause increasing concern in the Mediterranean Sea. In particular, special attention is now being paid to the ‘‘new pollution’’ processes; that is, the introduction of novel substances with biological activity that might have synergetic effects with ‘‘classical pollutants” [44]. The Patagonian Shelf, too, see increasing urban and industrial pollution and the contamination of coastal and marine environments. Harbors, marinas, seawalls, railway lines, and other structures on the seashore are common features in South Africa’s coastal cities [37]. Near- and off-shore pipelines continue to discharge increasing volumes of sewage, fish waste, or industrial effluent into the marine environment. Pollution is a serious threat in the Indian Ocean, where the main sources are industrial effluents (metals, oil, and organic byproducts) and nutrients from domestic sewage and agricultural runoff. Industrial pollutants act as direct toxic substances, causing impairment of metabolic functions and eventually mortality. The Indian Ocean Global Ocean Observing System [70] has conservatively estimated a daily release of 57 million liters of domestic sewage and 6 million liters of industrial sewage into the coastal waters of that ocean. Dead zones caused by eutrophication in the coastal waters have increased exponentially since 1960; of roughly 400 such zones recently cataloged [71], about 10 are in the Indian Ocean. Areas heavily affected by eutrophication and pollution in China are experiencing dramatic changes in biodiversity. In the Yellow Sea, only 7 of the 164 species recorded in 1967–68 were found in 1980s, and no living benthic animals were found after 1989. With the rapid development of industrial production in coastal cities and an increase in the intensity of fishing in the inner shelf area, and with a corresponding rise in environmental pollution and decline of living resources, the high biodiversity and richness of marine biota and living resources in China’s seas have seriously decreased. Chemical pollution and coastal nutrient and sediment input around New Zealand are low relative to that encountered in the more densely populated and industrialized countries of the Northern Hemisphere. Oil spills in New Zealand waters are usually minor but are not infrequent (e.g., 84 marine spills in 1996) and have caused losses of seabirds in affected areas. Other pollutants—thermal effluent, heavy metals, and radioactivity—have been likewise relatively small in quantity (some local exceptions) and biological impact [72]. Some of the principal pollution problems in New Zealand are due to the loss of nutrients from animal wastes (cattle numbers rose 34% between 1994 and 2002) and fertilizers into coastal waters. Raw sewage is pumped into the ocean with few exceptions. About 80% of marine pollution comes from land-based sources, and about three-quarters of this amount is discharged through outfalls. Industrial and urban wastes released at sea through storm-water runoff (including heavy metals) and the flushing of plastic and other debris to the coast are additional sources of pollution [73]. Plastic and other litter constitute a potential hazard to marine animals; plastic items are thought to cause more deaths of marine animals than oil spills, heavy metals, or other toxic materials. A year-long study of Auckland’s storm-water discharges found that each day 28,000 pieces of litter, much of it plastic, ended up in the Waitemata Harbour. In some coastal areas, reef sponges, kelp forests, weed beds, seagrasses, and fish nursery grounds have been lost because of increased sedimentation. Nutrient input from terrestrial runoff has been suggested as contributory to some harmful algal blooms (HABs), including red tides [74]. On some occasions, potent toxins produced by a small number of HAB species have been reported to cause either mass mortalities of marine life or to find their way from shellfish to humans, causing neurotoxic shellfish poisoning (NSP), paralytic shellfish poisoning (PSP), diarrhetic shellfish poisonings (DSP), and amnesic shellfish poisoning (ASP). Marine pollution in the Antarctic occurs mostly as a result of localized oil and sewage spills but is also caused by global pollutants from outside the Southern Ocean. Considering the extreme vulnerability of the biotic components and the persistence of pollutants in the system, these pollutants are likely to produce exacerbated effects.

In the Baltic Sea, the first signs of eutrophication were already evident in the mid-1950s, and this phenomenon has caused shrinkage of the distribution area and declines in the population of species preferring clear and oxygen-rich water. Other effects are impoverishment of species diversity, and increase in bioproductivity, and intensification of potentially toxic cyanobacterial blooms [75]. Reports of long-term decreases in oxygen concentrations at open ocean and coastal locations have prompted concern about the consequences for marine ecosystems [71]. In the Gulf of Mexico, extending hypoxic regions regularly drive mobile animals from certain areas, and increasing coastal development encroaches upon or destroys habitats [76]. In August 2006, an event with severe hypoxic and anoxic conditions on the central Oregon coast led to the complete absence of all fish from normally populated rocky reefs and high mortality of large benthic invertebrates [77]. In the Mediterranean Sea, hypoxic areas periodically appear in the shallow northern Adriatic Sea, causing extensive mortality of benthic organisms and bentho-nekton. It has been recently hypothesized that the sequence of jellyfish outbreaks, red tides, mucilage appearance, and bottom anoxia of the Adriatic Sea may all be part of a succession of interconnected events [78].

**Box 4. Examples of threats to marine biodiversity from alien species.**

The introduction of alien species is one of the major sources of pressure on Caribbean marine life [42, 79]. The invasive lionfish, *Pterois* spp., having no predators in this system and preying on small fishes, has an increasing impact on the endemic fish population of the southeastern United States and the Caribbean region. New species and assemblages are likely to be discovered in deep sponge and coral endofauna and in the complex hard bottom that underlies swift Gulf Stream currents on the Blake Plateau. Recent notable invasive species in the Gulf of Mexico include the Australian spotted jellyfish (*Phyllorhiza punctata*) and the brown and green mussels (*Perna perna* and *P. viridis,* respectively). The orange cup coral (*Tubastraea coccinea*), originally from the Indo-Pacific, invaded the Gulf of Mexico in the late 1970s and is now found throughout the area on oil and gas platforms, other artificial structures, and coral reefs [80]. Invasive species have been identified as an important cause of declining biodiversity in the Humboldt System. Although there are few reports of highly invasive or aggressive nonindigenous species in the Humboldt Current Large Marine Ecosystem [81], the introduction of non-native species represents a large risk to native biodiversity. The list of invasive species present in the Brazilian coasts includes 66 species [82] of phytoplankton (3), macroalgae (10), zooplankton (10), zoobenthos (38), fish (4), and pelagic bacteria (1), and has suffered increasing bioinvasion [82].

The mussel *Mytilus galloprovincialis* is a recognized invader around the world and has entered the Chilean coasts [82]. The presence of exotic species, in the Patagonian Shelf has grown in recent years to 41 species, including algae, molluscs, hydroids, bryozoans, ascidians, and crustaceans, with severe ecological and economic consequences [83, 84]. In Patagonia, the expansion of the *Undaria pinnatifida* (an invasive seaweed, whose growth is not controlled by the native sea urchins, [85]) is associated with a dramatic decrease in species richness and diversity of native seaweeds. In the Baltic, 110 alien species are now documented, and about 70 of them are known to have established self-reproducing populations (Baltic Sea Alien Species Database, 2009), and they include secondary introductions from both the North Sea and adjacent inland waters [86]. Most species come from the northwest Atlantic, the Ponto-Caspian region, and the western European waters. At least three potentially toxic alien species have been documented in the Baltic Sea [87]. In addition, the cryptogenic ship worm *Teredo navalis* is now fully established in the southwestern Baltic region. The occurrence of the alien ctenophore zooplankton predator, the American comb jelly *Mnemiopsis leidyi,* threatens cod egg abundance in the Baltic Sea [88].

The number of alien species documented in the Mediterranean is now over 500. This region has witnessed an overwhelming increase in the number of alien species since the beginning of the early nineteenth century, particularly due to the Suez Canal connecting the Mediterranean to the Red Sea and an increase in ship traffic [89]. *Mnemiopsis leidyi* has spread across the Mediterranean, from Israel to Spain in 2009. The ingression of a large number of allochthonous species of tropical origin is leading to what is called the “tropicalization” of the southern Mediterranean Sea and “meridionalization” of the northern sectors (increased proportion of indigenous thermophilic species; [90]). Tropical species have been entering the Mediterranean either through the Suez Canal (Lessepsian migration) or the Strait of Gibraltar for decades [91, 92], and in the last two decades, the number of tropical species that have also spread through the entire basin is growing. Examples of Erythrean aliens that crossed the Strait of Sicily include algae, a seagrass, many invertebrates, and fish [93-95]. The lack of (evidence of) species extinction coupled with establishment of alien species is apparently leading to an increased richness of the Mediterranean [96] . Among the alien species expected to pose serious problems to other species are the algae *Caulerpa taxifolia* [97] and *C. racemosa* var. *cylindracea* [98], the shellfish *Crassostrea gigas* and *Ruditapes philippinarum* (intentionally introduced for culture), the prawn *Fenneropenaeus merguiensis*, the scyphozoan jellyfish *Rhopilema nomadic*, and the striped catfish *Plotosus lineatus*. Several cases of poisoning and injuries due to an alien jellyfish (*Rhopilema nomadica*) have been reported from Israel [99]. *Cladocora caespitosa*, the most important among the shallow-water zooxanthellate species living in the Mediterranean [100] has suffered because of the warming episodes [101].

In South Africa there are 86 marine alien species and an additional 40 cryptogenic species, most of which are confined to sheltered sites (Mead et al. in prep). The most important introduced taxa in terms of numbers of species are the Crustacea (33 species), Mollusca (22 species), Ascidiacea (18 species), and Cnidaria (16 species). The Mediterranean mussel *Mytilus galloprovincialis* and the Pacific barnacle *Balanus glandula* have become widespread on the open, wave-exposed coastline. In the Pacific Hawaiian region, endemic species are showing limited resistance to the invasion of alien species. On the eastern Pacific coasts of California, the introduction and spread of alien species have emerged as major environmental, economic, and public health problems [102]. In San Francisco Bay, alien species dominate many important habitats [103]. Species such as the invasive tunicates *Didemnum* and *Styela clava*, European green crab, Japanese oyster drill, *Spartina* (cordgrasses), and various pathogens and parasites represent ongoing threats to the regional aquaculture industry.

## In the western Pacific the number of nonindigenous species is also increasing. At least 39 alien species have been recently introduced in Japanese waters, including 11 Mollusca, 10 each of the Annelida and Arthropoda, 3 Chordata, 2 Myxozoa, and 1 each of the Chlorophyta, Cnidaria, and Heterokontophyta. Some species, such as the gastropod *Nassarius sinarus,* have detrimental effects on the economy. Considering the importance if maritime traffic in Japan, that nation has a high rate of introduction of invasive species. The Chlorophyta *Caulerpa taxifolia* was also recently introduced into Japanese waters. One of the few countries to have successfully eradicated a marine invader is Australia [104], where the number of invasive marine species is continuously monitored. In New Zealand waters, the Asian kelp *Undaria pinnatifida* has been documented to produce negative effects on native species [105], but at least 200 non native marine species have become naturalized [106]. There are currently no records of successfully established invasive marine animal species within the Southern Ocean. There have been several reports of either adults or larvae of alien animals at Antarctic coastal localities, but none were found again or in more than one stage of their life cycle. The successfully established marine invaders found off the South Shetland Islands and the northern Antarctic Peninsula were benthic macroalgae [107].

## Box 5. Examples of how climate change threatens marine biodiversity.

A recent study of global patterns of the effects of climate change projected the distribution ranges of a sample of 1,066 exploited marine fish and invertebrates for 2050 using a newly developed dynamic bioclimate envelope model [108]. The study indicated that climate change could lead to many local extinctions in the subpolar regions, the tropics, and semienclosed seas and to more intense species invasions in the Arctic and the Southern Ocean. These projections suggest important species turnovers (>60% of the present biodiversity). These changes are likely to have negative consequences for ecosystem services, with important economic impact. In fact, climate change will cause a large-scale redistribution of global catch potential, with an average of 30%–70% increase in high-latitude regions and a drop of up to 40% in the tropics. Changes will be most apparent in the Pacific Ocean. An increase in catch potential is expected by 2055 in Norway, Greenland, and the Arctic, while significant losses could be observed in Indonesia, the United States (excluding Alaska and Hawaii), Chile, and China [109]. Warming will alter the geographic distribution of marine organisms, and such changes are expected to be particularly evident in regions characterized by psychrophilic species, such as the Arctic. Most of the present ice-covered areas are likely to have reduced ice cover, especially in summer, which could lead to increased primary and secondary production and possibly enhanced fish production [110]. Arctic benthic communities are likely to expand, displacing colder-water species. There will also be a shift northward in the distribution of many species of fishes, which could lead to extinction of some current Arctic species [111, 112]. Warmer temperatures in the Bering Sea, Chukchi Sea, and northeast coasts of the United States are causing a shift in the size composition of both planktonic and benthic assemblages with effects on the nekton [113-117]. Populations of zooplankton are getting smaller, while zooplankton predators such as chaetognaths are increasing and are having a significant impact on community organization [118]. Warmer temperatures are thought to be accompanied by earlier and higher zooplankton production [119].

These regions may experience a decline in benthic infaunal biomass. Major changes that are occurring in the benthic biomass and composition of species assemblages have consequences for the bird and mammal predators of those groups [120, 121]. A shift to pelagic production would favor bowhead, fin, minke, and blue whales but disadvantage benthic feeders such as gray whales, walrus, bearded seals, and diving ducks [120-122]. Surveys conducted since 1948 in the northeast Atlantic reported a peak in phytoplankton and zooplankton (*Calanus helgolandicus*) associated with a rise in temperature and Atlantic inflow in the late 1980s. On the basis of long-term observation, changes in climate and the North Atlantic Oscillation are expected to induce a regime shift [123] with simultaneous or time-lagged responses in biodiversity and ecosystem functioning. Cold-water species of the Yellow Sea and Japanese waters are now declining because of the rising sea temperature. The tellinid bivalve *Peronidia zyonoensis* Hatai et Nisiyama that was living 6,000 B.C. in the China Sea is now extinct, while living individuals could still be found in the northern Sea of Japan (Russian Far East) and east (Pacific) coast of Honshu, Japan. The Tropical East Pacific reefs are experiencing bleaching and a decrease of the live coral cover due to temperature increases of at least 1-2ºC associated to the El Niño-Southern Oscillation effect [124]. Climate change is likely to have an impact on marine ecosystems across the entire Hawaiian Archipelago, causing shifts in the abundance and distribution of marine species and increased incidence of harmful algal blooms and other nuisance species [102]. The temperature of the Mediterranean Sea has been steadily increasing since the 1970s [125, 126]. There are clues of rarefaction or even disappearance of cold-water species [56, 127, 128], starting from the deep-water coral *Lophelia pertusa* [129]. With global warming, thermophilic species of the southern Mediterranean are expanding northward [128, 130-132], while the abundance of some boreal species may dramatically decrease [133]. There are cases of species replacement [96, 127, 128, 134], and mass mortalities due to high temperature or development of pathogens [135, 136].

The decline of temperatures observed between 1987 and 2007 along the west and south coasts of Africa (Rouault pers. comm.), a result of climate-driven changes in upwelling patterns [137], is causing the declines in the warm-water indigenous brown mussel (*Perna perna*) and increases in kelp and the cold-water invasive mussel (*M. galloprovincialis*). Climate change is also apparently driving a strong movement in the center of gravity of both pelagic fish and rock lobster [138] stocks over the past decade, causing dramatic changes in the numbers of predatory seabirds [139]. There is increasing evidence of changes in the ranges of, the interactions between, native marine species under the influence of climate change also in Australia and New Zealand [104, 140, 141], and climate change is a significant potential threat to the long-term survival of Antarctic marine communities [142, 143]. The seas to the northeast and the west of the Antarctic Peninsula are some of the fastest-warming areas on Earth, showing a decrease of sea ice formation by 10% per decade in certain regions [144, 145]. The collapse of several floating ice shelves has dramatically altered coastal and shelf habitat on the peninsula. Because the frequency of ice scour on the shelf seabed is closely linked to sea ice duration, the catastrophic disturbance of shallow biodiversity is likely to significantly increase [146]. There has been an overall warming of surface waters (in the Bellingshausen and Scotia seas) by about 1°C in the last 50 years, but so far there is no evidence of any biologically meaningful temperature change in waters more than about 100 m deep.

The anthropogenic increase in the concentration of atmospheric carbon dioxide is reducing the pH of the oceans. Ocean acidification could have an impact on the prey base, fisheries species, and deep-water corals at high latitudes. In particular, calcification by bivalves that dominate polar shelves could be adversely affected, and thus the food web that relies on them [147]. Ocean acidification is also predicted to reduce the absorption of low-frequency sound, leading to a noisier environment for marine mammals [121, 148, 149]. A growing number of studies have demonstrated adverse impacts of acidification on marine organisms, including decreases in rates of coral calcification, reduced ability of algae and zooplankton to maintain protective shells, and reduced survival of larval marine shellfish and fish [121, 150, 151]. The southeast coasts of the United States along with the entire tropical region are expected to be highly sensitive to ocean acidification because of the large presence of calcifying organisms. The synergistic effects of temperature warming and ocean acidification on stony corals have been recently reviewed [152]. Acidification may be a major threat to Mediterranean marine biodiversity [153], affecting both calcifying phytoplankton (coccolithophores), which plays a significant role in the primary productivity of the oligotrophic Mediterranean Sea and has an impact on sessile organisms laying carbonate crusts. Calcareous red algae are key builders of coralligenous reefs in the Mediterranean, and sea water acidification is expected to impair their role [154]. Similar effects are expected for other oceanic regions at midlatitudes (such as the New Zealand) [155]. However, noncalcifying photosynthetic plants, such as frondose algae and seagrasses, may take advantage of a greater availability of CO_2_. The surface waters of the Southern Ocean are saturated with calcium carbonate. The Southern Ocean is predicted to be the first place where this acidification will reduce aragonite concentrations to below saturation point, by the year 2100 [148]. As the skeletons of the planktonic pteropod molluscs are aragonite based, it is unlikely that pteropods will be able to adapt quickly enough to survive in the Southern Ocean.

REFERENCES

1. Baum J, Myers R, Kehler D, Worm B, Harley S, et al. (2003) Collapse and conservation of shark populations in the Northwest Atlantic. Science 299: 389-392.
2. Frank K, Petrie B, Choi J, Leggett W (2005) Trophic cascades in a formerly cod-dominated ecosystem. Science 308: 1621-1623.
3. Myers R, Baum J, Shepherd T, Powers S, Peterson C (2007) Cascading effects of the loss of apex predatory sharks from a coastal ocean. Science 315: 1846-1850.
4. NMSP (2007) Florida Keys National Marine Sanctuary Revised Management Plan. Silver Spring, MD: U.S. Department of Commerce, National Oceanic and Atmospheric Administration, National Ocean Service, National Marine Sanctuary Program.
5. Coleman FC, Figueira WF, Ueland JS, Crowder LB (2004) The impact of United States recreational fisheries on marine fish populations. Science 305: 1958-1960.
6. Waters JR (2003) Review of the U.S. commercial red snapper fishery in the Gulf of Mexico. National Marine Fisheries Service & Southeast Fisheries Science Center, National Oceanic and Atmospheric Administration.
7. Ramírez-Rodriguez M, Chavez EA, Arreguín-Sánchez F (2000) Perspectives of the pink shrimp (*Farfantepenaeus duorarum* Burkenroad) fishery of Campeche Bank, Mexico. Cienc Mar 26: 97-112.
8. Arreguín-Sánchez F, Solís-Ramírez MJ, González de la Rosa ME (2000) Population dynamics and stock assessment for *Octopus maya* (Cephalopoda: Octopodidae) fishery in the Campeche Bank, Gulf of Mexico. Rev Biol Trop 48 (2/3): 323-331.
9. Tunnell JW Jr (1992) Natural versus human impacts to Southern Gulf of Mexico coral reef resources. In: Richmond RH, editor. Proceedings of the Seventh International Coral Reef Symposium. UOG Station, Guam: University of Guam Press. pp. 300-306.
10. Davies A, Wisshak M, Orr JC, Roberts JM (2008) Predicting suitable habitat for the cold-water coral *Lophelia pertusa* (Scleractinia). Deep-Sea Res Pt I 55: 1048-1062.
11. Wursig B, Jefferson TA, Schmidley DJ (2000) The Marine Mammals of the Gulf of Mexico. College Station, Texas: Texas A&M University Press. 232 p.
12. McKay M, Nides J, Lang W, Vigil D (2001) Gulf of Mexico Marine Protected Species Workshop, June 1999. New Orleans, LA: U.S. Dept of the Interior, Minerals Management Service, Gulf of Mexico OCS Region. 171 p.
13. Lindley ST, Grimes CB, Mohr MS, Peterson W, Stein J, et al. (2009) What caused the Sacramento River fall Chinook stock collapse? NOAA Technical Memorandum NOAA-TM-NMFS-SWFSC-447.
14. Kramer K, Heck KL (2007) Top-down trophic shifts in Florida Keys patch reef marine protected areas. Mar Ecol Prog Ser 349: 111-123.
15. Brewer D, Rawlinson N, Eayrs S, Burridge C (1998) An assessment of bycatch reduction devices in a tropical Australian prawn trawl fishery. [Fish Res](http://www.sciencedirect.com/science/journal/01657836) 36 (2-3): 195–215.
16. Brewer D, Heales D, Milton D, Dell Q, Fry G, et al. (2006) [The impact of turtle excluder devices and bycatch reduction devices on diverse tropical marine communities in Australia's northern prawn trawl fishery](http://www.sciencedirect.com/science?_ob=ArticleURL&_udi=B6T6N-4KN5JSY-2&_user=2322062&_coverDate=11%2F30%2F2006&_rdoc=1&_fmt=high&_orig=article&_cdi=5035&_sort=v&_docanchor=&view=c&_ct=26&_acct=C000056895&_version=1&_urlVersion=0&_userid=2322062&md5=f9abd9ac2736f86c3d45baa2f203ef66). Fish Res 81(2–3): 176–188.
17. Salini J, Brewer D, Farmer M, Rawlinson N (2000) [Assessment and benefits of damage reduction in prawns due to use of different bycatch reduction devices in the Gulf of Carpentaria, Australia](http://www.sciencedirect.com/science?_ob=ArticleURL&_udi=B6T6N-3Y6GVV9-1&_user=2322062&_coverDate=02%2F29%2F2000&_rdoc=1&_fmt=high&_orig=article&_cdi=5035&_sort=v&_docanchor=&view=c&_ct=26&_acct=C000056895&_version=1&_urlVersion=0&_userid=2322062&md5=d5426093f85741c7bde76e87600c727e). Fish Res 45(1): 1–8.
18. Robertson G, Williamson J, McNeill M, Candy SG, Smith N (2008) Autoliners and seabird by-catch: do line setters increase the sink rate of integrated weight longlines? CCAMLR Sci 15: 107–114.
19. Amorocho DF, Sánchez FA, Quiroga DD (2001) El encanto de las tortugas marinas en el Parque Nacional Natural Gorgona. In: Barrios LM, López-Victoria M, editors. Gorgona marina. Contribución al conocimiento de una isla única. Serie de Publicaciones Especiales No. 7, INVEMAR, Santa Marta, Colombia, 160 p.
20. Cañete, G., Bruno, C. & Copello, S. 2008. Estado actual de la actividad pesquera en el Mar Patagónico. In Estado de Conservación del Mar Patagónico y Áreas de Influencia. Puerto Madryn, Forum publication. Available: htpp://www.marpatagonico.org, pp. 163-186.
21. Defeo O, Horta S, Carranza A, Lercari D, de Alava A, et al. (2009) Hacia un manejo ecosistémico de pesquerías. Montevideo, Uruguay: Facultad de Ciencias - DINARA.
22. Orensanz JML, Schwindt E, Pastorino G, Bortolus A, Casas G, et al. (2002) No longer the pristine confines of the world ocean: a survey of exotic marine species in the southwestern Atlantic. Biol Invasions 4: 115-143.
23. López B, Pereira G (1998) Actualización del inventario de crustáceos decápodos del delta del Orinoco. In: López J, Saavedra L, Dubois M, editors. El río Orinoco. Aprovechamiento sustentable. Instituto de Mecánica de Fluidos. Memorias de las Primeras Jornadas Venezolanas de Investigación sobre el río Orinoco. 16 al 20 de Noviembre. Caracas: Universidad Central de Venezuela. pp: 76-86.
24. Molinet R, Arocha F, Cárdenas JJ (2008) Evaluación de los recursos pesqueros en el oriente venezolano. Petróleos de Venezuela, S.A. – Universidad Simón Bolívar, Caracas, Venezuela. 176 p.
25. Kaiser MJ, Ramsay K, Richardson CA, Spence FE, Brand AR (2000) Chronic fishing disturbance has changed shelf sea benthic community structure. J Anim Ecol 69(3): 494-503.
26. Kaiser MJ, Clarke KR, Hinz H, Austen MCV, Somerfield PJ, et al. (2006) Global analysis of response and recovery of benthic biota to fishing. Mar Ecol Prog Ser 311: 1-14.
27. Eero M, Köster FW, MacKenzie BR (2008) Reconstructing historical stock development of the eastern Baltic cod (*Gadus morhua*) before the beginning of intensive exploitation. Can J Fish Aquat Sci 65: 2728–2741.
28. Eero M, MacKenzie BR, Karlsdottir HM, Gaumiga R (2007). Development of international fisheries for cod (*Gadus morhua*) in the eastern Baltic Sea during 1880–1938. Fish Res 87: 155–166.
29. Lotze HK, Lenihan HS, Bourque BJ, Bradbury RH, Cooke RG et al. (2006) Depletion, degradation, and recovery potential of estuaries and coastal seas. Science 312(5781): 1806-1809.
30. Sardà F (1998) Symptoms of overexploitation in the stock of the Norway lobster (*Nephrops norvegicus*) on the" Serola Bank" (western Mediterranean Sea off Barcelona). Sci Mar 62(3): 295-299.
31. Thibaut T, Pinedo S, Torras X, Ballesteros E (2005) Long-term decline of the populations of Fucales (*Cystoseira* spp. and *Sargassum* spp.) in the Alberes coast (France, North-western Mediterranean). Mar Pollut Bull 50(12): 1472-1489.
32. Antoniadou C, Vafidis D (2008) First assessment of *Microcosmus sabatieri* (Tunicata: Ascidiacea) small-scale fishery in the South Aegean Sea (Eastern Mediterranean). Cah de Biol Mar 49: 97-100.
33. De la Rosa J, Saiz JI, Moreno D (2008) *Sipunculus nudus* Linnaeus, 1767 Tita. In: Barea JM, Ballesteros E, Moreno D, editors. Libro Rojo de los Invertebrados de Andalucia. Sevilla: Junta de Andalucía. pp. 639-642.
34. Pronzato R, Manconi R (2008) Mediterranean commercial sponges: over 5000 years of natural history and cultural heritage. Mar Ecol-Evol Persp 29(2): 146-166.
35. Penny AJ, Mann-lang JB, Van der elst RP, Wilke CG (1999) Long-term trends in catch and effort in the Kwazulu-Natal nearshore linefisheries. SA J Mar Sci 21: 51-76.
36. Griffiths MH (2000) Long term trends in catch and effort of commercial linefish off South Africa’s Cape Province: Snapshots of the 20th Century. S Afr J Mar Sci 22: 81-110.
37. Griffiths CL, Van Sittert L, Best PB, Brown AC, Clark BM, et al. (2004) Human impacts on marine animal life in the Benguela – a historical overview. Oceanogr Mar Biol: Ann Rev 42: 303-392.
38. Qasim SZ, Wafar MVM, Sumitra-Vijayaraghavan, Royan JP, Krishnakumari L (1978) Biological productivity of the coastal waters of India – from Dahbol to Tuticorin. Indian J Mar Sci 7: 84-93.
39. ACIA (2005) Arctic Climate Impact Assessment. Cambridge: Cambridge University Press. 1042 p.
40. Anderson JB (2007) The Formation and Future of the Upper Texas Coast. Texas, College Station: Texas A&M University Press. 163 p.
41. Gardner TA, Côté IM, Gill JA, Grant A, Watkinson AR (2003) Long-term region wide declines in Caribbean coral reefs. Science 301: 958–960.
42. Agard JBR, Cropper A (2007) Caribbean Sea Ecosystem Assessment report. Carib. Mar. Stud. (J. Inst. Mar. Affairs Trinidad & Tobago), Special Edition, 85 p.
43. Díaz JM, Gómez DI (2003) Cambios históricos en la distribución y abundancia de praderas de pastos marinos en la Bahía de Cartagena y áreas aledañas (Colombia). Bol Invest Mar Cost 32: 57-74.
44. Danovaro R (2003) Pollution threats in the Mediterranean Sea: An Overview. Chem Ecol 19 15-32.
45. Danovaro R, Pusceddu A (2007) Ecomanagement of biodiversity and ecosystem functioning in the Mediterranean Sea: concerns and strategies. Chem Ecol 23 (5): 347-360.
46. Wafar S, Untawale AG (2001) Mangroves. In: Sen Gupta R, Desa E, editors. The Indian Ocean. A perspective. Taylor & Francis. pp. 539-561.
47. Mazlan AG, Zaidi CC, Wan-Lofti WM, Othman BHR (2005) On the current status of coastal marine biodiversity in Malaysia. Indian J Mar Sci 34: 76-87.
48. Hutomo M, Moosa MK (2005) Indonesian marine ands coastal biodiversity: Present status. Indian J Mar Sci 34: 88-97.
49. Stefani M, Vincenzi S (2005) The interplay of eustasy, climate and human activity in the late Quaternary depositional evolution and sedimentary architecture of the Po Delta system. Mar Geol 222: 19-48.
50. Airoldi L, Beck MW (2007) Loss, status and trends for coastal marine habitats of Europe. Oceanogr Mar Biol 45: 345-405.
51. Montefalcone M, Morri C, Peirano A, Albertelli G, Bianchi CN (2007) Substitution and phase shift within the *Posidonia oceanica* seagrass meadows of NW Mediterranean Sea. Estuarine Coastal and Shelf Science 75(1-2): 63-71.
52. Tudela S (2004) Ecosystem effects of fishing in the Mediterrean [Mediterranean]: an analysis of the major threats of fishing gear and practices to biodiversity and marine habitats. General Fisheries Council for the Mediterranean Studies and Reviews 74 2004: i-vi, 1-44.
53. IUCN (2009) The IUCN redlist of threatened species (International Union for Conservation of Nature). Data available at: <http://www.iucnredlist.org/>.
54. Guidetti P, Terlizzi A, Fraschetti S, Boero F (2003) Changes in Mediterranean rocky-reef fish assemblages exposed to sewage pollution. Mar Ecol-Prog Ser 253: 269-278.
55. Sala E (2004) The past and present topology and structure of Mediterranean subtidal rocky-shore food webs. Ecosystems 7(4): 333-340.
56. Boero F, Bonsdorff E (2007) A conceptual framework for marine biodiversity and ecosystem functioning. Mar Ecol-Evol Persp 28: 134-145.
57. Coll M, Lotze HK, Romanuk TN (2008) Structural degradation in Mediterranean Sea food webs: Testing ecological hypotheses using stochastic and mass-balance modelling. Ecosystems 11(6): 939-960.
58. Roberts JM, Wheeler AJ, Freiwald A (2006) Reefs of the deep: The biology and geology of cold-water coral ecosystems. Science 312: 543-547.
59. Jensen A, Frederiksen R (1992) The fauna associated with the bank-forming deepwater coral *Lophelia pertusa* (Scleractinaria) on the Faroe shelf. Sarsia 77: 53-69.
60. Mortensen PB, Hovland M, Brattegard T, Farestveit R (1995) Deep water bioherms of the scleractinian coral *Lophelia pertusa* (L.) at 64° N on the Norwegian shelf: structure and associated megafauna. Sarsia 80: 145-158.
61. Husebø Å, Nøttestad L, Fosså JH, Furevik DM, Jørgensen SB (2002) Distribution and abundance of fish in deep-sea coral habitats. Hydrobiologia 471: 91-99.
62. Costello MJ, McCrea M, Freiwald A, Lundalv T, Jonsson L, et al. (2005) Functional role of deep-sea cold-water *Lophelia* coral reefs as fish habitat in the north-eastern Atlantic. In: Freiwald A, Roberts JM, editors. Cold-water corals and ecosystems. Berlin Heidelberg: Springer Verlag. pp. 771-805.
63. Harding KC, Härkönen TJ (1999) Development in the Baltic grey seal (Halichoerus grypus) and ringed seal (*Phoca hispida*) populations during the 20th century. Ambio 28: 619–627.
64. Calabretta C, Oviatt C (2008) The response of benthic macrofauna to anthropogenic stress in Narragansett Bay, Rhode Island: A review of human stressors and assessment of community conditions. Mar Pollut Bull 56: 1680-1695.
65. Seitz R, Dauer D, Llansó R, Long W (2009) Broad-scale effects of hypoxia on benthic community structure in Chesapeake Bay, USA. J Exp Mar Biol Ecol 381: S4-S12.
66. Gulf.of.Mexico.Alliance (2006) Governor’s Action Plan for Healthy and Resilient Coasts. Gulf of Mexico Alliance. 32 p.
67. Cruz M, Gabor N, Mora E, Jiménez R, Mair J (2003) The known and unknown about marine biodiversity in Ecuador (continental and insular). Gayana 67(2): 232-260.
68. Zapata FA, Vargas-Ángel B, Garzón-Ferreira J (2001) Salud y conservación de las comunidades coralinas. In Barrios L, López-Victoria M, editors. Gorgona marina, contribución al conocimiento de una isla única. INVEMAR, Serie de Publicaciones Especiales No. 7, Santa Marta, Colombia. pp. 41-50.
69. Klein E, Cárdenas JJ, Esclasans D (2009) Prioridades de conservación de la biodiversidad marina del Frente Atlántico y Golfo de Paria. Proyecto LOCTI. INTECMAR – The Nature Conservancy. Technical Report.
70. IOGOOS (2002) An Indian Ocean observing strategy. Strawman for the first IOGOOS meeting, Mauritius, p. 16.
71. Diaz RJ, Rosenberg R (2008) Spreading dead zones and consequences for marine ecosystems. Science 321: 926–929.
72. Ridgway NM, Glasby GP (1984) Sources of marine pollution around New Zealand. NZOI Oceanogr Summ 23: 1–21.
73. Hutching G, Walrond C (2009). Marine conservation. Te Ara – the Encyclopedia of New Zealand. Available: http://www.TeAra.govt.nz/EarthSeaAndSky/OceanStudyAndConservation/Marine Conservation/en
74. Rhodes LL, Adamson JE, Rublee P, Schaeffer E (2006) Geographic distribution of *Pfiesteria piscicida* and *P. shumwayae* (Pfiesteriaceae) in Tasman Bay and Canterbury, New Zealand (2002–2003). NZ J Mar Freshw Res 40: 211–220.
75. HELCOM (2009) Eutrophication in the Baltic Sea – An integrated thematic assessment of the effects of nutrient enrichment and eutrophication in the Baltic Sea region. Balt Sea Environ Proc No 115B. Helsinki: HELCOM. 148 p.
76. Rabalais N (2004) Hypoxia in the Gulf of Mexico. In: Withers K, Nipper M, editors. Environmental Analysis of the Gulf of Mexico. Texas: Harte Research Institute for Gulf of Mexico Studies. pp. 478-489.
77. Chan F, Barth JA, Lubchenco J, Kirincich A, Weeks H, et al. (2008) Emergence of anoxia in the California Current Large Marine Ecosystem. Science 319: 920.
78. Boero F (2001) Adriatic ecological history: a link between jelly outbreaks, red tides, mass mortalities, overfishing, mucilages, and thaliacean plakton? In: Brian F, editor. Gelatinous plankton outbreaks: theory and practice. Monaco: CIESM Workshop Series. pp. 44-46.
79. [Burke](http://www.wri.org/profile/lauretta-burke) L, Maidens J (2004) Reefs at risk in the Caribbean. Washington: World Resources Institute. 80 p.
80. Fenner D, Banks K (2004) Orange cup coral *Tubastraea coccinea* invades Florida and the Flower Garden Banks, northwestern Gulf of Mexico. Coral Reefs 23: 505-507.
81. Castilla JC, Neill PE (2009). Marine Bioinvasions in the Southeastern Pacific: Status, Ecology, Economic Impacts, Conservation and Management. In: Rilov G, Crooks J, editors. Marine Bioinvasions: Ecology, Conservation, and Management Perspectives. Springer. pp. 439-458.
82. Ferreira CEL, Junqueira AOR, Villac MC, Lopes RM (2008) Marine bioinvasions in the Brazilian Coast: brief report on history of events, vectors, ecology, impacts and management of non-indigenous species. In: Rilov G, Crooks JA, editors. Biological Invasions in Marine Ecosystems. Berlin: Springer-Verlag. pp. 459-478.
83. Bigatti G, Penchaszadeh PE (2008) Invertebrados del Mar Patagónico, diagnóstico de la problemática actual y potencial de su conservación y manejo. In Estado de Conservación del Mar Patagónico y Áreas de Influencia [on-line]. Puerto Madryn, Forum publication, available at: htpp://www.marpatagonico.org, pp. 105-133.
84. Schwindt E. 2008. Invertebrados del Mar Patagónico, diagnostico de la problemática actual y potencial de su conservación y manejo. In Estado de Conservación del Mar Patagónico y Áreas de Influencia [on-line]. Puerto Madryn, Forum publication, available at: htpp://www.marpatagonico.org, pp. 274-302.
85. Teso SV, Bigatti G, Casas GN, Piriz ML, Penchaszadeh PE (2009) Do native grazers from Patagonia, Argentina, consume the invasive kelp *Undaria pinnatifida*? Rev Museo Arg de Ciencias Naturales ns 11 (1): 7-14.
86. Leppäkoski E, Gollasch S, Gruszka P, Ojaveer H, Olenin S, Panov V (2002) The Baltic - a sea of invaders. Can J Fish Aquatic Sci 59: 1209–1228.
87. Hajdu S, Pertola S, Kuosa H (2005) *Prorocentrum minimum* (Dinophyceae) in the Baltic Sea: morphology, occurrence - a review. Harmful Algae 4: 471–480.
88. Haslob H, Clemmesen C, Schaber M, Hinrichsen H-H, Schmidt JO et al. (2007) Invading *Mnemiopsis leidyi* as a potential threat to Baltic fish. Mar Ecol Progr Ser 349: 303-306.
89. Coll M, Piroddi C, Kaschner K, Ben Rais Lasram F, Steenbeek J, Aguzzi J, et al. (2010) The biodiversity of the Mediterranean Sea: estimates, patterns, and threats. PLoS ONE. In press.
90. Bianchi CN, Morri C (2003) Global sea warming and “tropicalization” of the Mediterranean Sea: biogeographic and ecological aspects. Biogeographia 24: 319-327.
91. Por FD (1978) Lessepsian migrations: the influx of Red Sea biota into the Mediterranean by way of the Suez Canal. Heidelberg: Springer. 228 p.
92. Galil BS (2006) The Suez Canal - The marine caravan - The Suez Canal and the Erythrean invasion. In: Gollasch S, Galil bS, Cohen AN, editors. Monographiae Biologicae: Bridging divides: maritime canals as invasion corridors. Heidelberg: Springer. pp. 207-300.
93. Gambi MC, Barbieri F, Bianchi CN (2008) New record of the alien seagrass *Halophila stipulacea* (Hydrocharitaceae) in the western Mediterranean: a further clue to changing Mediterranean Sea biogeography. JMBA2 Biodiversity Records: 7.
94. Garibaldi F, Orsi Relini L (2008) Record of the bluespotted cornetfish *Fistularia commersonii* Rüppell, 1838 in the Ligurian Sea (NW Mediterranean). Aquatic Invasions 3(4): 471-474.
95. Galil BS (2009) Taking stock: inventory of alien species in the Mediterranean Sea. Biological Invasions 11(2): 359-372.
96. Galil BS (2007) Loss or gain? Invasive aliens and biodiversity in the Mediterranean Sea. Marine Pollution Bulletin 55(7-9): 314-322.
97. Meinesz A, Simberloff D, Quammen D (2002) Killer Algae. Chicago : University of Chicago Press. 360 p.
98. Verlaque M, Afonso-Carrillo J, Candelaria Gil-Rodríguez M, Durand C, Boudouresque C et al. (2004) Blitzkrieg in a marine invasion: *Caulerpla racemosa var.* *cylindracea* (Bryopsidales, Chlorophyta) reaches the Canary Islands. Biological Invasions 6(269-281).
99. Galil BS (2007) Seeing red: Alien species along the Mediterranean coast of Israel. Aquatic Invasions 2(4): 281-312.
100. Peirano A, Morri C, Bianchi CN, Aguirre J, Antonioli F et al. (2004) The Mediterranean coral *Cladocora caespitosa*: a proxy for past climate fluctuations? Global and Planet Change 40(1-2): 195-200.
101. Rodolfo-Metalpa R, Richard C, Allemand D, Bianchi CN, Morri C et al. (2006) Response of zooxanthellae in symbiosis with the Mediterranean corals *Cladocora caespitosa* and *Oculina patagonica* to elevated temperatures. Mar Biol 150: 45-55.
102. Risien J (2009) West Coast Regional Marine Research and Information Needs. Corvallis: Oregon Sea Grant. 56 p.
103. Cohen AN, Carlton JT (1998) Accelerating Invasion Rate in a Highly Invaded Estuary. Science 279: 555-558.
104. Bax NJ, Hayes K, Marshall A, Parry D, Thresher R (2002) Man-made marinas as sheltered islands for alien marine organisms: establishment and eradication of an alien invasive marine species. In: Veitch CR, Clout MN, editors. Turning the tide: the eradication of invasive species. Auckland Invasive Species Specialist Group of the World Conservation Union (IUCN) Occasional Paper 27. Gland, Switzerland: IUCN. pp. 26–29.
105. Russell LK, Hepburn CD, Hurd CL, Stuart MD (2008) The expanding range of *Undaria pinnatifida* in southern New Zealand: distribution, dispersal mechanisms and the invasion of wave-exposed environments. Biol Invasions 10: 103–115.
106. Gould B, Ahyong ST (2008) Marine Invasives Taxonomic Service. MAF Biosecurity NZ 85: 18–19.
107. Clayton MN, Wiencke C, Klöser H (1997) New records and sub-Antarctic marine benthic macroalgae from Antarctica. Polar Biol 17: 141–149.
108. Cheung WWL, Lam VWY, Sarmiento JL, Kearney K, Watson R, et al. (2009) Projecting global marine biodiversity impacts under climate change scenarios. Fish Fish 10: 235-251.
109. Cheung WWL, Lam VWY, Sarmiento JL, Kearney K, Watson R, Zeller D, Pauly D (2010) Large-scale redistribution of maximum fisheries catch potential in the global ocean under climate change. Glob Change Biol 16: 24-35.
110. Leong H (2005) Marine Systems. In: Symon C, Arris L, Heal B, editors. ACIA, Arctic Climate Impact Assessment 2005: Cambridge: Cambridge University Press. pp. 453-538.
111. Sirenko BI, Gagaev SY (2007) Unusual abundance of macrobenthos and biological invasions in the Chukchi Sea. Russ J Mar Biol 33: 355-364.
112. Usher MB (2005) Principles of Conserving the Arctic’s Biodiversity. In: Symon C, Arris L, Heal B, editors. ACIA, Arctic Climate Impact Assessment 2005: Cambridge: Cambridge University Press. pp. 539-596.
113. Fogarty MJ, Incze LS, Hayhoe K, Mountain D, Manning J (2008) Potential climate change impacts on Atlantic cod (*Gadus morhua*) off the northeastern USA. Mitig Adapt Strat Global Change 13: 453-466.
114. Nixon S, Granger S, Buckley B, Lamont M, Rowell B (2004) A one hundred and seventeen year coastal water temperature record from Woods Hole, Massachusetts. Estuaries 27: 397-404.
115. Greene C, Pershing A (2007) OCEANS: Climate Drives Sea Change. Science 315: 1084-1085.
116. Collie J, Wood A, Jeffries H (2008) Long-term shifts in the species composition of a coastal fish community. Can J Fish Aquat Sci 65: 1352-1365.
117. Nye J, Link J, Hare J, Overholtz W (2009) Changing spatial distribution of fish stocks in relation to climate and population size on the Northeast United States continental shelf. Mar Ecol Prog Ser 393: 111-129.
118. Hunt GL, Jr., Stabeno PJ, S. Strom, Napp JM (2008) Patterns of spatial and temporal variation in the marine ecosystem of the southeastern Bering Sea, with special reference to the Pribilof Domain. Deep-Sea Res Pt II 55: 1919-1944.
119. Hunt Jr. GL, Stabeno P, Walters G, Sinclair E, Brodeur RD, et al. (2002) Climate change and control of the southeastern Bering Sea pelagic ecosystem. Deep-Sea Res Pt II 49: 5821-5853.
120. Grebmeier JM, Cooper LW, Feder HM, Sirenko BI (2006) Ecosystem dynamics of the Pacific-influenced northern Bering and Chukchi seas in the Amerasian Arctic. Prog Oceanogr 71: 331-361.
121. Hester KC, Peltzer ET, Kirkwood WJ, Brewer PG (2008) Unanticipated consequences of ocean acidification: A noisier ocean at lower pH. Geophys Res Lett 35: L19601.
122. Piepenburg D (2005) Recent research on Arctic benthos: common notions need to be revised. Polar Biol 28: 733-755.
123. Reid PC, Edwards M (2001) Long-term changes in the pelagos, benthos and fisheries of the North Sea. Senckenb Marit 32: 107–115.
124. Díaz JM (2000) Áreas Coralinas de Colombia. Publ. Esp. Instituto de Investigaciones Marinas y Costeras (INVEMAR), 5, Santa Marta, Colombia, 172 p.
125. Salat J, Pascual J (2002) The oceanographic and meteorological station at l'Estartit (NW Mediterranean). In: Brian F, editor. Tracking long-term hydrological change in the Mediterranean Sea Mediterranean Science Commision Ciesm Workshop Series, nº 16. Monaco. pp. 29-32.
126. Féral JP (2008) Are climate changes already threatening sessile species (or species with low mobility) in the North-Western Mediterranean Sea? Vulnerability of coastal ecosystems. In: Briand F, editor. Climate warming and related changes in Mediterranean marine biota CIESM Workshop Monographs. Monaco. pp. 79-87.
127. Chevaldonné P, Lejeusne C (2003) Regional warming-induced species shift in NW Mediterranean marine caves. Ecol Lett 6: 371-379.
128. Sabatés A, Martín P, Lloret J, Raya V (2006) Sea warming and fish distribution: the case of the small pelagic fish, *Sardinella aurita*, in the western Mediterranean. Glob Change Biol 12(11): 2209-2219.
129. Zibrowius H (1980) Les Scléractiniaires de la Méditerranée et de l’Atlantique nord-orientale. Mémoires de l’Institut Océanographique, Monaco 11: 1-284.
130. Astraldi M, Bianchi CN, Gasparini GP, Morri C (1995) Climatic fluctuations, current variability and marine species distribution: a case study in the Ligurian Sea (north-west Mediterranean). Oceanol Acta 18(2): 139-149.
131. Bianchi CN, Morri C (2000) Marine biodiversity of the Mediterranean Sea: situation, problems and prospects for future research. Mar Pollut Bull 40(5): 367-376.
132. Goren M, Galil BS (2005) A review of changes in the fish assemblages of Levantine inland and marine ecosystems following the introduction of non-native fishes. J Appl Ichthyol 21(4): 364-370.
133. Quignard JP, Raibaut A (1993) Ichthyofauna of the languedocian coast (gulf of Lion) faunistic and demographic modification. Vie et milieu 43(4): 191-195.
134. Morri C, Bianchi CN (2001) Recent changes in biodiversity in the Ligurian Sea (NW Mediterranean): is there a climatic forcing? In: Faranda FM, Guglielmo L, Spezie G, editors. Structure and processes in the Mediterranean ecosystems: Milan: Springer. pp. 375-384.
135. Cerrano C, Bavestrello G (2009) Mass Mortalities and Extinctions. In: Wahl M, editor. Marine hard bottom communities ecological studies 206. Berlin: Springer-Verlag. pp. 295-303.
136. Cerrano C, Bavestrello G, Bianchi CN, Cattaneo-Vietti R, Bava S, et al. (2000) A catastrophic mass-mortality episode of gorgonians and other organisms in the Ligurian Sea (Northwestern Mediterranean), summer 1999. Ecol Lett 3(4): 284-293.
137. Snyder MAL, Sloan C, Diffenbaugh NS, Bell JL (2003) Future climate change and upwelling in the California Current. Geophys Res Lett 30: 1823, doi:10.1029/2003GL017647.
138. Cockcroft AC, van Zyl D, Hutchings L (2008).Large-scale changes in the spatial distribution of South African West Coast rock lobster: an overview. Afr J Mar Sci 30: 149-159.
139. Crawford RJM, Sabarros PS, Fiarweather T, Underhill LG,Wolfaardt AC (2008). Implications for seabirds off South Africa of a long-term change in the distribution of sardine. Afr J Mar Sci 30: 177-184.
140. Condie SA, Harris PT (2005) Interactions between physical, chemical, biological and sedimentological processes in Australia’s shelf seas. In: Robinson AR, Brink KH, editors. The Sea. Volume 14. Cambridge, Mass: Harvard College, Harvard University Press. pp. 1413–1449.
141. Ross DJ, Johnson CR, Hewitt CL, Ruiz GM (2004) Interaction and impacts of two introduced species on a soft-sediment marine assemblage in SE Tasmania. Mar Biol 144: 747–756.
142. Clarke A, Murphy EJ, Meredith MP, King JC, Peck LS, et al. (2007) Climate change and the marine ecosystem of the western Antarctic Peninsula. Phil Trans R Soc B 362: 149–166. (doi:101098/rstb20061958)
143. Barnes DKA, Peck LS (2008) Vulnerability of Antarctic shelf biodiversity to predicted regional warming. Clim Res 37: 149-16.
144. Meredith MP, King JC (2005) Rapid climate change in the ocean west of the Antarctic Peninsula during the second half of the 20th century. Geophys Res Lett 32: L19604. (doi:101029/2005GL024042).
145. Whitehouse MJ, Meredith MP, Rothery P, Atkinson A, Ward P, et al. (2008) Rapid warming of the ocean around South Georgia Southern Ocean during the 20th century: forcings characteristics and implications for lower trophic levels. Deep-Sea Res Pt I 55: 1218-1228.
146. Smale DA, Barnes DKA, Fraser KPP, Peck LS (2008) Benthic community response to iceberg scouring at an intensely disturbed shallow water site at Adelaide Island Antarctica. Mar Ecol-Prog Ser 355: 85-94.
147. Grebmeier JM, Barry JP (2007) Benthic processes in polynyas. In Smith WO Jr, Barber DG. Polynyas: windows to the World's oceans. Amsterdam, The Netherlands: Elsevier. pp. 363-390.
148. Orr JC, Fabry VJ, Aumont O, Bopp L, Doney SC, et al. (2005) Anthropogenic ocean acidification over the twenty-first century and its impact on calcifying organisms, Nature 437, 681-686.
149. Talmage S, Gobler C (2009) The effects of elevated carbon dioxide concentrations on the metamorphosis, size, and survival of larval hard clams (*Mercenaria mercenaria*), bay scallops (*Argopecten irradians*), and Eastern oysters (*Crassostrea virginica*). Limnol Oceanogr 54: 2072-2080.
150. Gazeau F, Quiblier C, Jansen JM, Gattuso JP, Middelburg JJ, et al. (2007) Impact of elevated CO2 on shellfish calcification. Geophys Res Lett 34: L07603 (doi:10.1029/2006GL028554).
151. Dextrase AJ, Mandrak NE (2006) Impacts of alien invasive species on freshwater fauna at risk in Canada. Biol Invasions 8: 13-24.
152. Hoegh-Guldberg O, Mumby PJ, Hooten AJ, Steneck RS, Greenfield P, et al. (2007) Coral reefs under rapid climate change and ocean acidification. Science 318, 1737-1742.
153. Hall-Spencer JM, Rodolfo-Metalpa R, Martin S, Ransome E, Fine M, et al. (2008) Volcanic carbon dioxide vents show ecosystem effects of ocean acidification. Nature 454(7200): 96-99.
154. Kuffner IB, Andersson AJ, Jokiel PL, Rodgers KS, Mackenzie FT (2008) Decreased abundance of crustose coralline algae due to ocean acidification. Nature Geoscience 1(2): 114-117.
155. Smith AM (2009) Bryozoans as southern sentinels of ocean acidification: a major role for a minor phylum. Mar Freshw Res 60: 475–482.
